# Molecular Dynamics Analysis of Inhibitor Binding Interactions in the *Vibrio cholerae* Respiratory Complex NQR

**DOI:** 10.1002/prot.70036

**Published:** 2025-09-29

**Authors:** Joseph. A. DePaolo‐Boisvert, Karina Tuz, David. D. L. Minh, Oscar X. Juarez

**Affiliations:** ^1^ Department of Chemistry Illinois Institute of Technology Chicago Illinois USA; ^2^ Department of Biology Illinois Institute of Technology Chicago Illinois USA

**Keywords:** antibacterial agents, molecular dynamics simulation, NAD(P)H dehydrogenase (quinone), *Vibrio cholerae*

## Abstract

The sodium‐pumping ubiquinone oxidoreductase sodium pumping quinone reductase (NQR) is an important enzyme in the respiratory chain of multiple pathogenic gram‐negative bacteria. NQR has been proposed as a viable antibiotic target due to its importance in supporting energy‐consuming reactions and its absence in human cells. In this study, molecular dynamics simulations were conducted to characterize the interactions between the ubiquinone binding pocket of *Vibrio cholerae* NQR with its substrate analogue ubiquinone‐4 and three potent inhibitors: HQNO, aurachin‐D42, and korormicin‐A. Through interaction fingerprinting, distance calculations, and clustering analysis, important binding motifs for each of these ligands were identified. Subunit B residues K54, F137, E144, V145, V155, E157, G158, F159, and F160 were frequently identified as establishing either hydrogen bonding interactions or hydrophobic interactions with these three ligands. The findings of this in silico study are interpreted in view of mutagenesis analyses previously published in the literature. The elucidation of important binding interactions associated with the inhibitors is critical as it informs structure–activity relationships, which are essential for the development of novel antibiotics targeting NQR.

## 1 Introduction

One of the biggest global challenges in public health of our time is antibiotic resistance. Clinical isolates of pathogens, including bacteria, have increasingly shown antibiotic resistance mechanisms. Every year, antimicrobial resistance is directly responsible for 1.27 million deaths and contributes to nearly 5 million deaths worldwide [1]. This problem is exacerbated by the lack of both new antibiotics and new targets. In this study, we focus on a new target—the sodium pumping quinone reductase (NQR) complex, a critical enzyme in the respiratory chain of multiple gram‐negative pathogenic bacteria, including *Vibrio cholerae* , *Pseudomonas aeruginosa, Klebsiella* bacteria, and *Chlamydia trachomatis* [2, 3, 4, 5, 6, 7, 8, 9, 10, 11, 12, 13, 14]. Analogously to mitochondrial complex I, NQR catalyzes electron transfer from NADH to Ubiquinone, feeding the lower part of the respiratory chain [2, 3, 10, 15, 16]. Electron transfer drives the pumping of sodium ions from the cytoplasm to the periplasm, establishing a gradient that is essential for energy‐consuming reactions, such as ATP synthesis, pH regulation, rotation of the flagellar motor, drug efflux, ion homeostasis, and nutrient transport [3, 4, 8, 17, 18, 19, 20]. Moreover, NQR activity regulates virulence factor production [21]. Due to the importance of NQR in bacterial physiology, especially in its ability to produce disease, and its absence in human cells, it has been proposed as a viable antibiotic target [6, 22, 23, 24].

**FIGURE 1:**
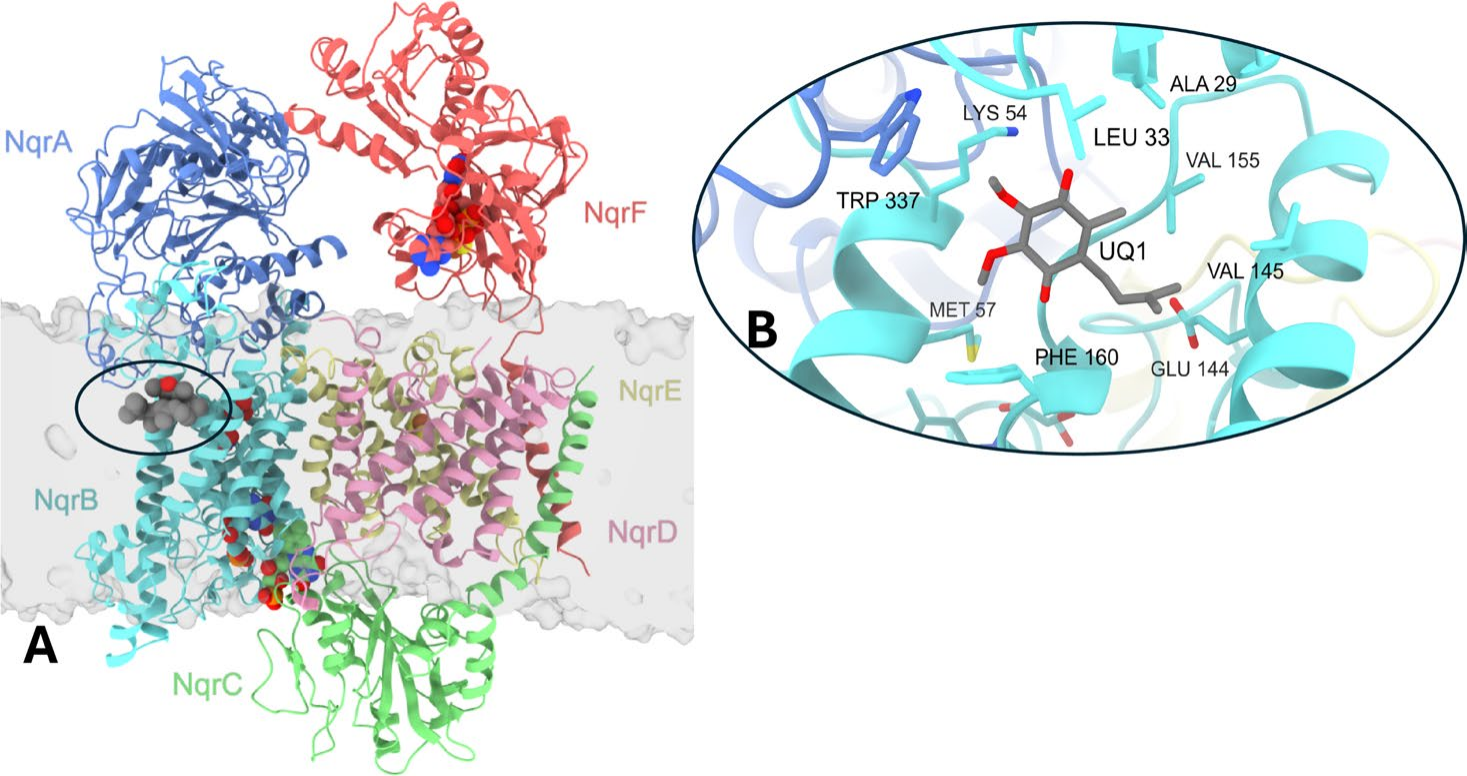
Initial pose of ubiquinone‐4 simulations. (A) Structure of *Vibrio cholerae* NQR with ubiquinone‐4 (gray) in complex. Cofactors FAD, FMN, riboflavin, and iron–sulfur clusters are also shown as spheres. NqrA: blue, NqrB: cyan, NqrC: green, NqrD: pink, NqrE: yellow, NqrF: orange. (B) Close‐up view of the ubiquinone binding pocket of this study with the isoprenoid chain of ubiquinone‐4 trimmed to one unit for clarity.

NQR is composed of six subunits (A–F) and carries six redox cofactors, four of which are covalently bound to the enzyme (Figure 1). Subunit A is entirely cytosolic, and subunit F carries a cytosolic domain and one transmembrane crossing. The cytosolic domains of subunit F also bear FAD and 2Fe‐2S_F_ cofactors. These cofactors are the first two members of the electron transfer pathway; FAD accepts electrons from NADH, which are then transferred to 2Fe‐2S_F_ [2, 9]. Subunits B, D, and E are multi‐transmembrane helix proteins and carry most of the remaining cofactors. The 2Fe‐2S_DE_ center is found in subunits D and E, in a pocket located in the core of the membrane. This cofactor appears to be relevant for electron transport, but its participation has not been confirmed experimentally [15, 25]. Subunit C carries a single membrane crossing and a periplasmic domain with one covalently bound FMN, whose reduction is associated with the capture of a cytosolic sodium ion [26]. Following the reduction of this cofactor, electrons move to another FMN molecule in subunit B, and subsequently riboflavin, which is embedded in the core of subunit B [27]. Ultimately, electrons are delivered to ubiquinone, reducing it to ubiquinol.

Previous studies have identified several molecules that act as inhibitors of NQR, including the naturally produced compounds HQNO (2‐heptyl‐4‐quinolinol 1‐oxide), korormicin‐A [28], and aurachin‐D42 [9], as well as the synthetic inhibitor PEG‐2S [22], a korormicin‐A analogue. The tridimensional structure of *V. cholerae* NQR has been obtained at high resolution, and recent work has been able to identify the binding site of HQNO, aurachin‐D, and korormicin‐A in Cryo‐EM structures deposited in the Protein Data Bank as 8A1Y [29], 7XK6 [9], and 7XK7 [9], respectively. The inhibitors are bound in a membrane‐embedded pocket comprised mainly of subunit B but also with the participation of subunit A, where a ubiquinone substrate binding site has been located (PDB: 8A1U, 8A1V, and 8A1W by Hau et al. [29]) (Figure 1). Our recently deposited Cryo‐EM structures 8EVU and 8EW3 also corroborate that this pocket carries a tightly bound ubiquinone, which has been reported in previous studies [2, 30]. NQR contains two ubiquinone sites, the target pocket of this study, as well as a catalytically active site embedded in a cleft of membrane subunits B and D [30]. However, the exact function of these two sites has not yet been elucidated.

While there are a multitude of NQR structures determined by Cryo‐EM and X‐ray crystallography, a comprehensive molecular dynamics analysis of the critical interactions between NQR and these inhibitors has not been performed. To develop new antibiotics against this enzyme, it is paramount to understand how inhibitors bind to the enzyme. This study identifies the critical ligand‐protein interactions of the substrate analogue ubiquinone‐4, as well as the inhibitors HQNO, aurachin‐D42, and korormicin‐A with the ubiquinone binding site located in subunits NqrA and NqrB (Figure 1). Our results show that hydrophobic residues NqrB‐F137, NqrB‐V145, NqrB‐V155, NqrB‐F159, and NqrB‐F160 establish contacts with multiple ligands. Moreover, residues NqrB‐K54, NqrB‐E144, and NqrB‐G158 establish hydrogen bonds, and residues NqrB‐F160 and NqrA‐W337 transiently participate in π‐stacking interactions with the inhibitors. This study allows the identification of critical residues that stabilize inhibitor and substrate binding to this site, providing critical information to define binding modes, which can be utilized in the development of novel antibiotics targeting NQR using the methods described here.

## 2 Methods

### 2.1 Preparation

Models of *V. cholerae* NQR were built from PDB structures 8EVU and 8ACY based on the Orientations of Proteins in Membranes Database [31]. Because subunit F was not fully resolved in 8EVU, MDAnalysis 2.7.0 [32, 33] was used to align Subunit F from 8ACY onto 8EVU [34]. The 8ACY structure was aligned to the 8EVU from the Orientations of Proteins in Membranes Database [31]. In both models, the protein was protonated at pH 7 with PDB2PQR 3.6.2 [35, 36, 37]. The organic cofactors requiring protonation (FAD, RBF, and 2 FMNs) were protonated in their fully oxidized states with AmberTools 23.6 [38]. The forcefield parameters for the protein and cofactors were replicated from previous studies [2, 27, 39, 40] and can be obtained on github [41]. The environment (water, salt, membrane) was created using the CHARMM‐GUI membrane builder tool [42, 43, 44, 45, 46, 47, 48, 49]. A heterogeneous membrane composed of 1‐palmitoyl‐2‐oleoyl‐sn‐glycero‐3‐phosphoethanolamine (POPE), 1‐palmitoyl‐2‐oleoyl‐sn‐glycero‐3‐phosphoglycerol (POPG), and cardiolipin in a 84:10:4 ratio [50] was used for both the upper and lower leaflets. A salt concentration of neutralizing 150 millimolar sodium chloride was used. As in previous studies, the lipid17 [27] and OPC3 [51] force fields were used to parameterize the membrane, water, and salt [2, 27]. However, these force fields lack parameters for cardiolipin. Parameters for the doubly anionic form of cardiolipin were generated using the Amber GAFF2 force field, following charge assignment by the AM1‐BCC method with antechamber [38, 52].

Models of NQR‐ligand complexes were prepared based on the PDB structure 8EVU. Four ligands were prepared for this study: ubiquinone‐4, HQNO, korormicin‐A, and aurachin‐D42 (Supporting Figure S1). SMILES strings for these four ligands were obtained from PubChem [53]. Each SMILES string was converted to PDB format using the OpenFF toolkit 0.16.6 [54]. PDB files of the protein (without cofactors) and the four ligands were converted to PDBQT format using MGLTools 1.5.7 [55]. Each of the four ligands was then docked to the ubiquinone site of the crystal structure (8EVU) using AutoDock Vina [56, 57]. The best scoring poses of ubiquinone‐4, HQNO, and aurachin‐D42, which closely resemble crystal poses, were used for the initial structure of molecular dynamics (MD) simulations. However, no docking poses of korormicin‐A recapitulated known crystal poses. For this reason, the crystal pose of korormicin‐A from structure 7XK7 was used by aligning protein residues within 20 Å of korormicin‐A to the same residues of 8EVU. Each ligand was assigned charges with the AM1‐BCC method of antechamber and parameterized with the Amber GAFF2 force field [38, 52]. Throughout the preparation process, UCSF ChimeraX 1.8 was used for visualization [58, 59, 60].

### 2.2 Simulation

Systems were simulated using OpenMM 8.1 [61]. A series of relaxation steps, similar to our previous study [2], was used—minimization; 250 ps of constant volume simulation (NVT) with restraints on protein heavy atoms and the Z coordinate of membrane heavy atoms; 250 ps of NVT with no restraints; 2.5 ns of constant pressure simulation (NPT) with an XY‐anisotropic Monte Carlo‐Membrane Barostat; 2.5 ns of NPT with a Monte Carlo‐Barostat. Following the relaxation steps, production simulations of each system were performed in triplicate for at least 500 ns. All simulations were run with the Langevin integrator [62] (300 K temperature, 1/ps friction coefficient, 2 fs timestep), and frames recorded to disk every 10 ps.

### 2.3 Analysis

The 13 production simulations were primarily analyzed with the MDAnalysis 2.7.0 [32, 33] and ProLIF 2.0.3 [63] Python packages. In the analysis, residues will be described with a code describing their identity, number, and chain/subunit in the format Nqr(ChainID)‐(Single Letter Code) (Residue Number), i.e., NqrB‐A29. Simulations based on 8ACY were analyzed only for dynamics (root mean square deviation [RMSD] and root mean square fluctuation [RMSF]) and no ligand binding experiments were carried out.

#### 2.3.1 RMSD and RMSF of Atomic Positions

The whole protein was aligned by the alpha carbons to the first frame of the production simulation. RMSD values were calculated for all alpha carbons as well as alpha carbons of each chain. RMSF values were calculated based on the best alignments of chains onto themselves; each chain being aligned by the alpha carbons to the first frame of the production simulation.

#### 2.3.2 Binding Pose Clustering

Trajectories were aggregated by ligand and aligned by all atom alignment of residues that have any atom within 20 Å of the ligand. The ligand coordinates were extracted and the HDBScan method of scikit‐learn 1.5.2 [64] was used with a precomputed distance matrix (the heavy atom RMSD of the ligand coordinates). A slice of every tenth available frame was used for this clustering due to memory limitations. The medoid of each cluster was computed by determining the cluster member with the lowest average RMSD to all other cluster members.

#### 2.3.3 Interaction Determination and Fingerprinting

ProLIF was used to analyze the presence of interactions between the protein and ligand for every tenth frame of all trajectories. Results were aggregated by ligand and reported as the mean and standard deviation (*n* = 3) of the percentage of simulation frames in which interactions were present. Interactions occurring in less than 10% of simulation frames were not plotted.

#### 2.3.4 Hydrogen Bond Distance Analyses

Distances relevant to all hydrogen bonding interactions reported by the ProLIF analysis were calculated. Trajectories were aligned analogously to *Binding Pose Clustering*. ProLIF identified the residues with which a ligand is interacting, but visualization was necessary to determine if hydrogen bonding occurs via the side chain or backbone of a residue. In cases where there are multiple potential interacting atoms on a residue's side chain (e.g., acceptance of a hydrogen bond by a GLU side chain), the reported distance is between the closest relevant atom of the ligand and the closest relevant atom of the side chain. Otherwise, the distance between the closest relevant ligand atom (oxygen in the case of acceptance, hydroxyl oxygen or nitrogen in the case of donation) and the relevant protein atom was calculated.

#### 2.3.5 NqrB‐F160 Distance and Phase Angle Analyses

Distances were computed between the center of mass of the heavy atoms of the NqrB‐F160 side chain and the center of mass of the heavy atoms of the aromatic warhead of ligands. The phase angle was computed by selecting three atoms each from the ligand warheads and NqrB‐F160 side chain to define the plane that each ring resides in. The reported phase angle is the dot product of vectors normal to the two planes (NqrB‐F160 and the ligand aromatic warhead). Korormicin‐A, while lacking an aromatic warhead, was included in this analysis by considering the furan‐like ring as the warhead.

## 3 Results

### 3.1 Molecular Dynamics Simulations

**FIGURE 2:**
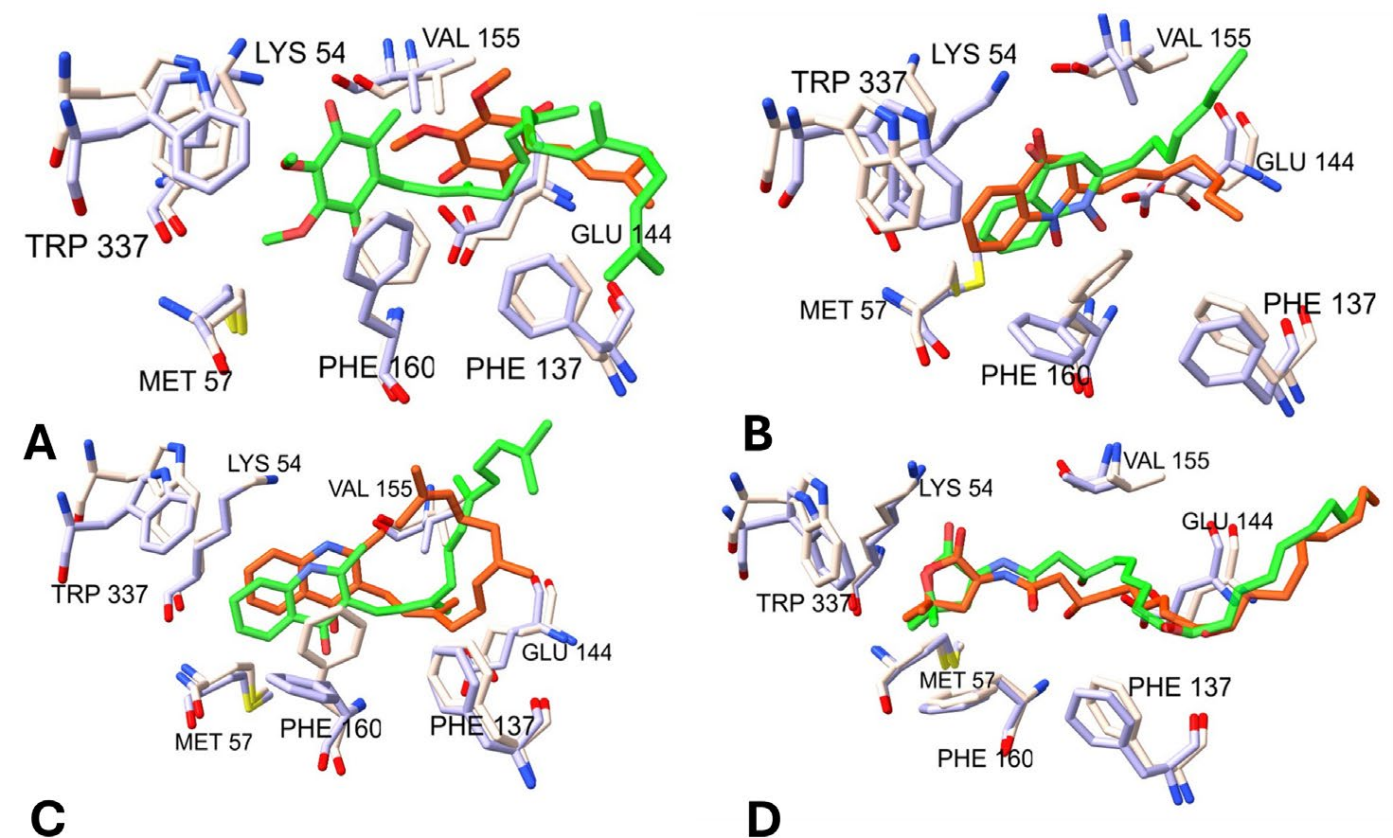
Comparison of simulation cluster medoids to crystal poses of *Vc* NQR. (A) ubiquinone‐4—simulation: lavender protein and green ligand; 8A1W: peach protein and orange ligand. (B) HQNO—simulation: lavender protein and green ligand; 8A1Y: peach protein and orange ligand. (C) Aurachin‐D42—simulation: lavender protein and green ligand; 7XK6: peach protein and orange ligand. (D) Korormicin‐A—simulation: lavender protein and green ligand; 7XK7: peach protein and orange ligand.

MD simulations complement cryo‐EM and X‐ray crystallography by providing the range of motion, conformational flexibility, and dynamics of biomolecules, which are entirely missing in static structure methods. While static structures can binarily classify the existence of atomic‐scale interactions, the functional motions captured during MD are critical for quantifying these interactions. MD allows for the detailed analysis of ligand‐binding events, revealing transient interactions and induced conformation changes that could not be inferred from a static structure. In this work, we carried out MD simulations of *V. cholerae* NQR embedded in a homogenous membrane, similar to that found physiologically in cells, containing POPE, POPG, and Cardiolipin in a physiologic 84:10:4 ratio [50]. The simulations were carried out using neutralizing 150 mM NaCl at pH 7.0. Ubiquinone‐4, HQNO, aurachin‐D42, and korormicin‐A were simulated in the NqrB ubiquinone binding pocket near the membrane solvent interface (Figure 1), where these inhibitors have been reported to be bound [9, 29]. MD simulations were run for 500 ns with a 2 fs timestep at 300 K. Our results show that the dominant pose of simulations is highly analogous to the experimentally determined structures found in recent Cryo‐EM data (Figure 2), demonstrating consistency between the molecular mechanics force field and experimental observations and opening the possibility to extend these studies to other undiscovered inhibitors.

**FIGURE 3:**
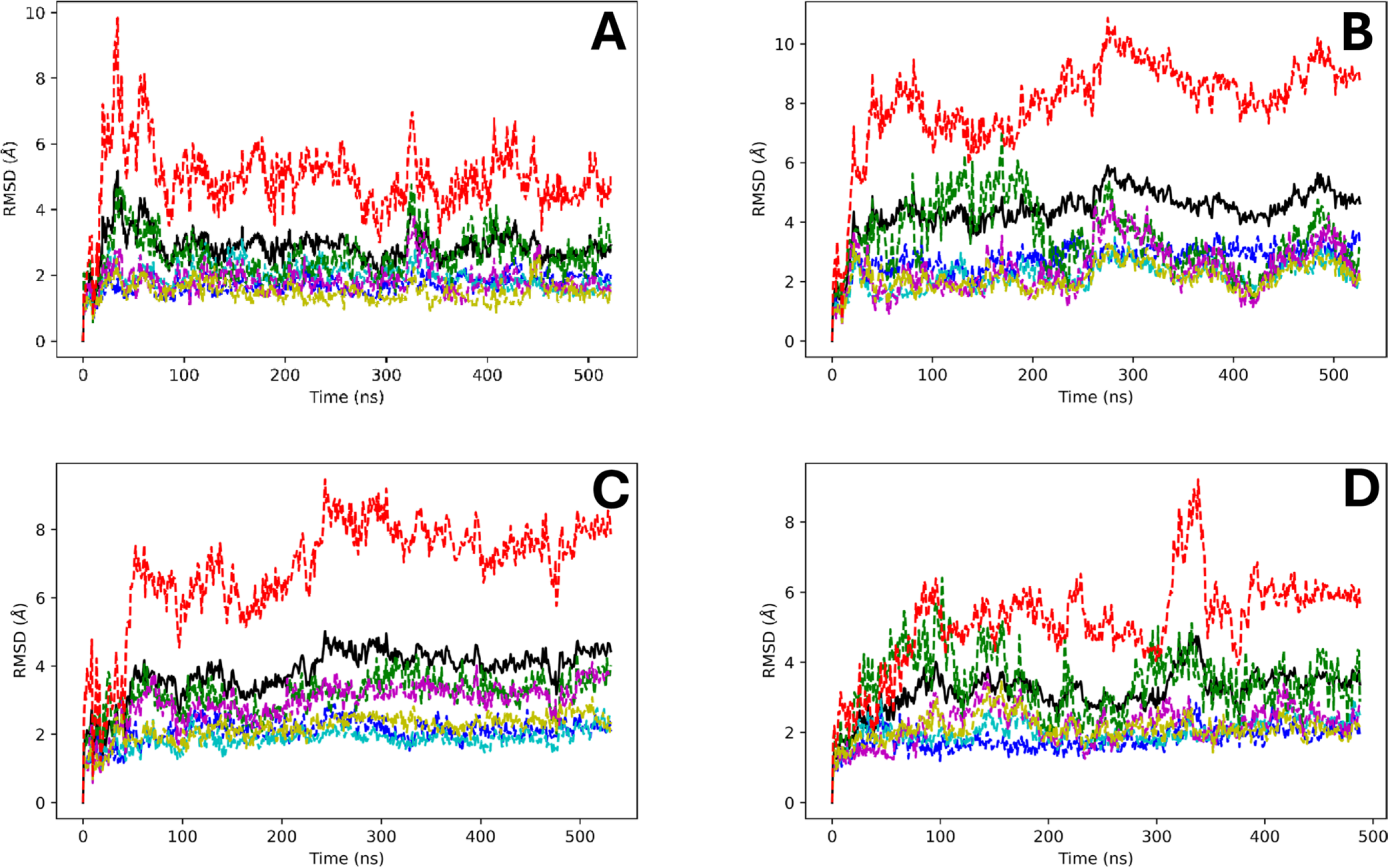
Root mean square deviations of NQR alpha carbons’ positions during select simulations. Black: all alpha carbon average, dark blue: NqrA, light blue: NqrB, green: NqrC, magenta: NqrD, yellow: NqrE, red: NqrF. (A) Ubiquinone‐4, (B) HQNO, (C) Aurachin‐D42, and (D) korormicin‐A.

**FIGURE 4:**
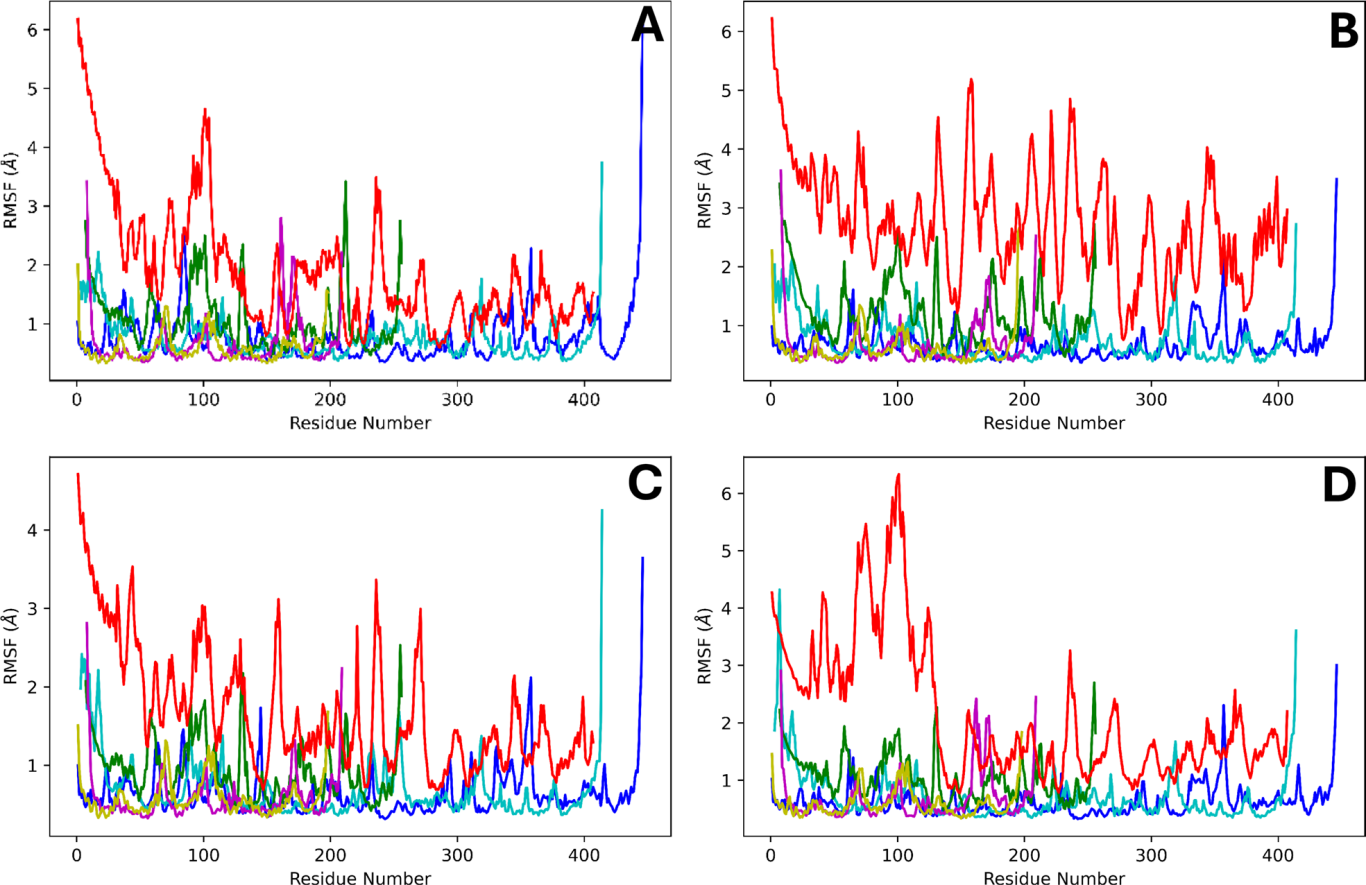
Root mean square fluctuations (RMSF) of alpha carbons during select simulations. Dark blue: NqrA, light blue: NqrB, green: NqrC, magenta: NqrD, yellow: NqrE, red: NqrF. (A) Ubiquinone‐4, (B) HQNO, (C) Aurachin‐D42, and (D) korormicin‐A.

In our simulations, subunit NqrF was the most dynamic chain by RMSD and RMSF analysis (Figures 3 and 4, Supporting Figure S2A–D), with chain‐specific RMSD from the first frame ranging from 6 to 12 Å in different simulations. The membrane‐embedded chains NqrB, NqrD, and NqrE were least dynamic in all simulations, with RMSD of 2–3 Å and RMSF of 1–2 Å. While there is considerable variation across triplicate simulations of the three inhibitors (HQNO, aurachin‐D42, and korormicin‐A), on average the RMSD is 1–1.5 Å higher than that of the ubiquinone‐4 simulation, typically due to greater RMSD associated with chain NqrF. To validate our results, in particular regarding the flexibility of subunit F, an MD simulation was carried out with the published 8ACY structure, which was solved with all subunits in the complex. The data show that the 8ACY MD simulation has similar RMSD values for each chain as observed in simulations of 8EVU with Ubiquinone‐4 (Figure 3, Supporting Figure S2A–E), albeit the RMSF values are markedly lower. High RMSD is attributable to concerted domain motions of the FAD and 2Fe‐2S binding around the hinge regions. Interestingly, subunit NqrC was observed as more dynamic in this validation simulation than in many of our production simulations.

### 3.2 Interactions of Ligands and Inhibitors With Binding Sites

**FIGURE 5:**
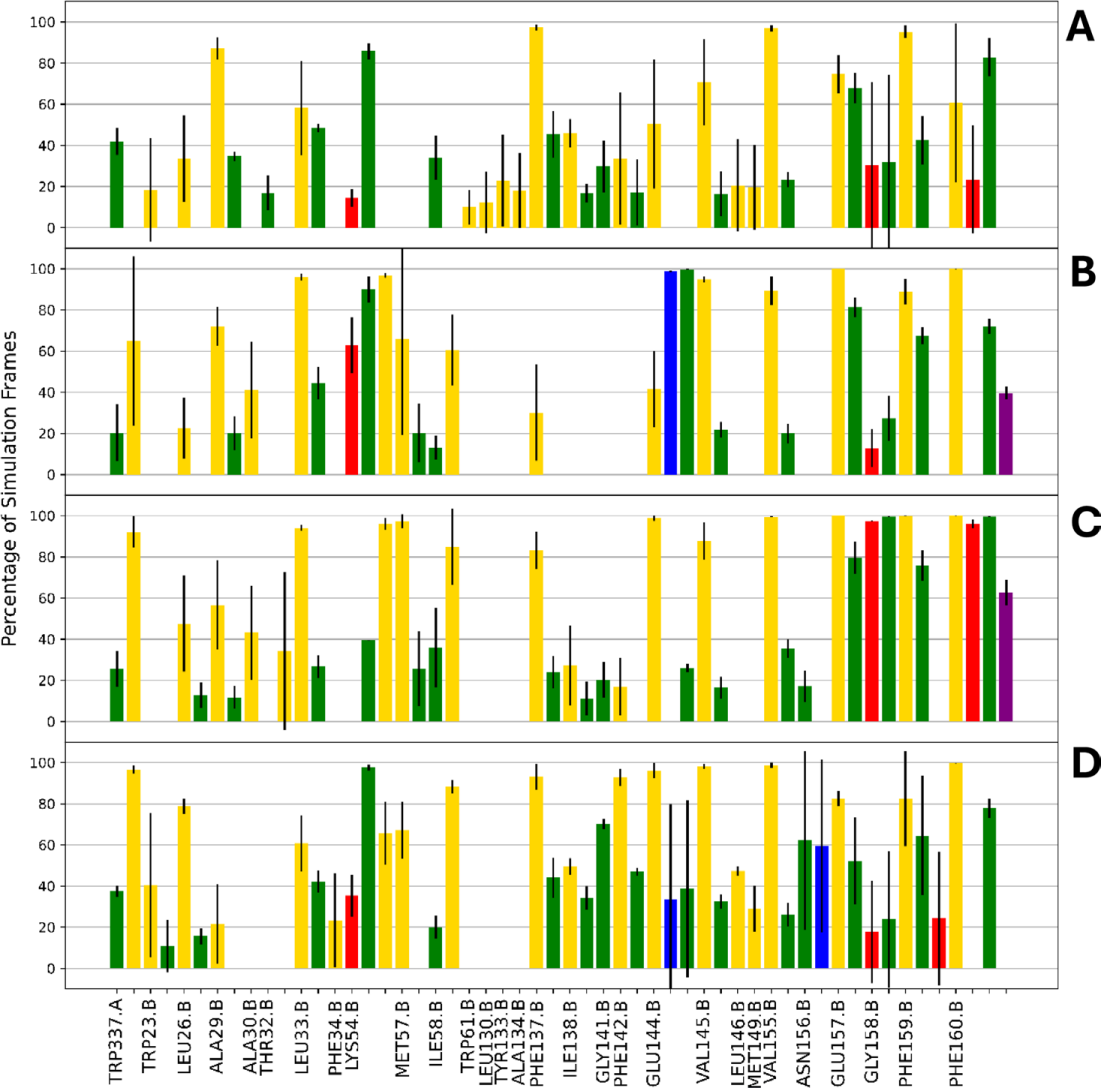
Mean and standard deviation (*n* = 3) of percentage of simulation frames where interactions between NQR residues and ligands are present. Interactions with a mean < 10% are not shown. H‐bond acceptor/donor indicates that the ligand is the acceptor/donor. Unlabeled x‐ticks refer to the residue labeled to the left. Yellow: hydrophobic, green: Van der Waals, red: H‐bond acceptor, blue: H‐bond donor, purple: π‐stacking. (**A**) Ubiquinone‐4, (**B**) HQNO, (**C**) Aurachin‐D42, and (**D**) Korormicin‐A.

**FIGURE 6:**
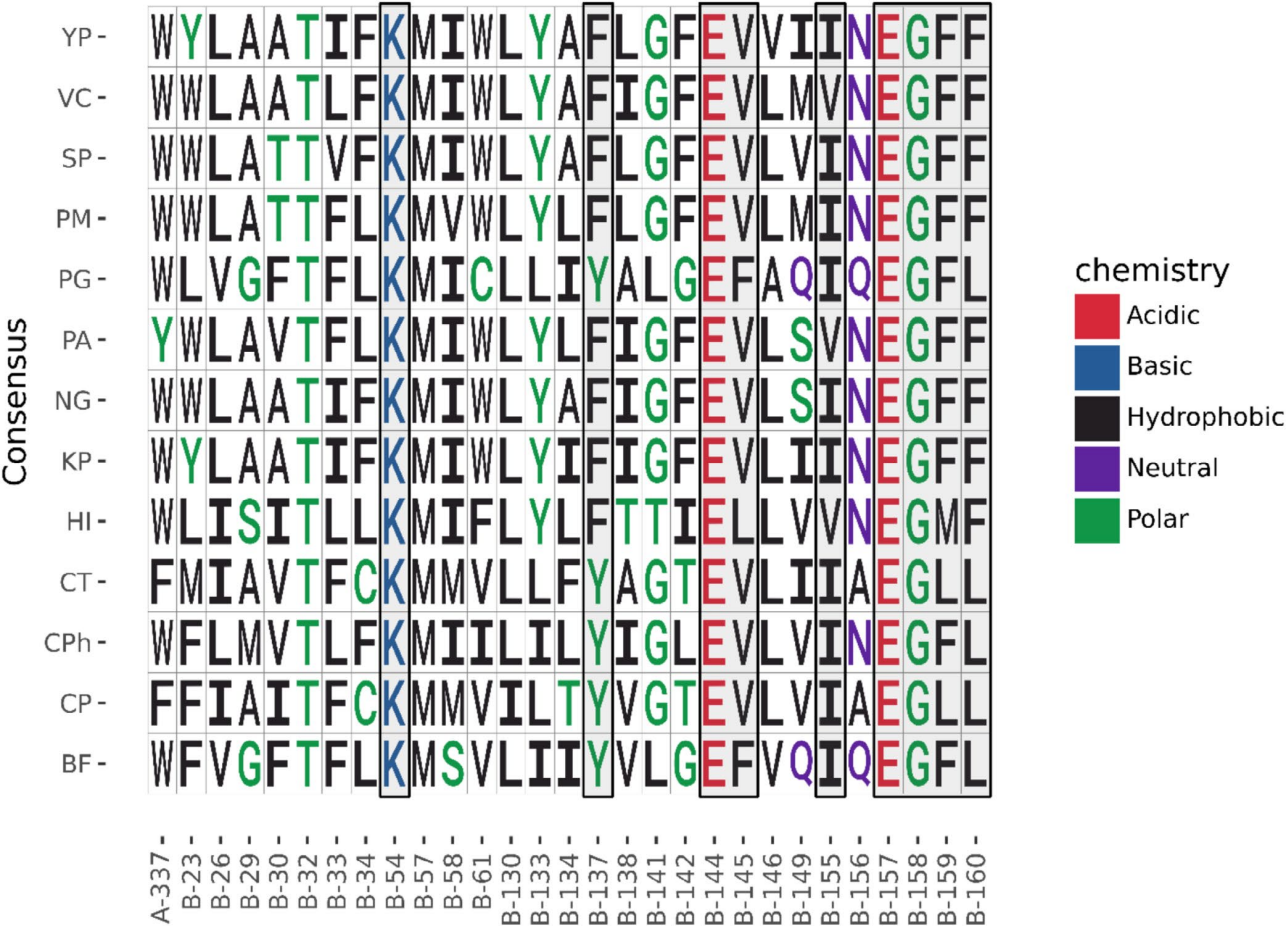
Sequence Analysis of residues identified by ProLIF. BF, *Bacteroides fragilis* ; CP, *Chlamydia pneumoniae* ; CPh, *Chlorobium pheobacteroides*; CT, *Chlamydia trachomatis* ; HI, *Haemophilus influenza*; KP, *Klebsiella pneumoniae* ; NG, *Neisseria gonorrhoeae* ; PA, *Pseudomonas aeruginosa* ; PG, *Porphyromonas gingivalis* ; PM, *Pasteruella mutocida*; SP, *Serratia proteomaculans*; VC, *Vibrio cholerae* ; YP, *Yersinia pestis* .

ProLIF analyses were performed to characterize hydrophobic and Van der Waals contacts (VdW) of the protein with the inhibitors. ProLIF defines hydrophobic interactions as a hydrophobic ligand atom and a hydrophobic protein atom being within 4.5 Å of each other, and a VdW contact as a ligand atom and a protein atom being within a distance of the sum of their VdW radii (with a 0.6 Å tolerance) [63]. Averaging (*n* = 3) over simulations with the same ligand, hydrophobic interactions were detected in more than 80% of simulation frames between all ligands and residues NqrB‐K54, NqrB‐V155, NqrB‐G158, NqrB‐F159, and NqrB‐F160 (Figure 5). For at least any three ligands, residues NqrB‐F137, NqrB‐E144, NqrB‐V145, and NqrB‐E157 also established hydrophobic interactions with the ligand. Of these residues, NqrB‐K54, NqrB‐E144, NqrB‐E157, and NqrB‐G158 are completely conserved across the sequences of a diverse set of 13 bacteria (Figure 6). Residues NqrB‐G158, NqrB‐F159, and NqrB‐F160 were found to form an important triad of hydrogen bonding residues. All ligands established a hydrogen bond accepting interaction to one or more of these residues via their respective backbone nitrogen (Figure 5). Hydrogen bonding to these residues is also relevant for the establishment of π‐stacking interactions with NqrB‐F160, as the combination of these helps to lock the head of all ligands in the binding pocket. Although π‐stacking with NqrB‐F160 was detected by ProLIF for HQNO and aurachin‐D42, but not for ubiquinone‐4, our own distance and phase angle calculations (Supporting Figure S3A–D) between NqrB‐F160 and the aromatic warheads of ubiquinone‐4, HQNO, and aurachin‐D42 demonstrate that this interaction is also likely. ProLIF defines a face‐to‐face π‐stacking interaction as one where aromatic rings are located within 4.5 Å of each other, and vectors normal to the interacting rings have an angle less than 40°. An edge‐to‐face π‐stacking interaction is defined as one where aromatic rings are located within 6.0 Å of each other, and vectors normal to the interacting rings have an angle greater than 50°. In simulations of ubiquinone‐4, HQNO, and aurachin‐D42, the distance and angle between the aromatic ligand warhead and NqrB‐F160 meet the criterion for ProLIF. The π‐stacking interaction between NqrB‐F160 and ubiquinone‐4 is therefore simply not reported by ProLIF since the ubiquinone‐4 warhead is not in fact aromatic.

NqrB‐K54 was also identified as a promiscuous hydrogen bond donor via the R‐group for all ligands other than aurachin‐D42, which bears an H‐bond donating group in the analogous position of accepting groups on ubiquinone‐4 and HQNO. Distance calculations corroborate the interaction determined by ProLIF (Supporting Figure S4A,B,D) and indicate that the side‐chain nitrogen of NqrB‐K54 was very infrequently farther than 1 Å from the relevant hydrogen bonding distance. NqrB‐E144 and NqrB‐N156 were also found to accept a hydrogen bond from the ligands (via the R‐group for E144, and the backbone for N156). Visualization and distance analysis (Supporting Figure S4E) show that these two residues fulfill a highly similar function in the establishment of H‐bonding, as interaction with either of these residues is exclusive and the relevant accepting atoms occupy the same position in the binding pocket surface. Of the hydrogen bonding and π‐stacking residues, NqrB‐K54 and NqrE‐144 both establish interactions via their side‐chain groups and are completely conserved across a diverse set of 13 pathogenic bacteria (Figure 6). The residues NqrB‐N156, NqrB‐G158, NqrB‐F159, and NqrB‐F160 are also highly conserved, albeit these established hydrogen bonding interactions via their backbones.

## 4 Discussion

Our recently deposited Cryo‐EM structures 8EVU and 8EW3, together with those of others (8A1U [29], 8A1V [29], 8A1W [29], 8A1Y [29], 7XK6 [9], and 7XK7 [9]) demonstrate that the target pocket of this study both carries a tightly bound ubiquinone and is the binding site of multiple potent NQR inhibitors. In this work, MD simulations of ubiquinone‐4, HQNO, aurachin‐D42, and korormicin‐A were carried out bound to NQR for the first time in this binding pocket in order to better understand the interactions between these substrates/inhibitors and NQR. Subunit NqrF, which was added from 8ACY to our structure (8EVU) displays high levels of dynamics by RMSD and RMSF analysis. In the validation simulation of 8ACY, similar RMSDs of NqrF were observed as in our simulations with ubiquinone‐4, although the RMSF values were significantly lower. The much lower RMSF of NqrF in the simulation of 8ACY indicates that the domains of NqrF move much more concertedly and with less fluctuation of individual residues about their average position, but similar overall motion of the subunit. In visualization of our simulations, NqrF domains exhibited both conformational changes and hinge motions that were not observed in the simulation of 8ACY. These results are consistent with our Cryo‐EM data showing that subunit F is highly flexible, and indeed we were not able to solve subunit F in our structure. Thus, the MD simulations found here represent the likely behavior of this subunit in the membrane environment.

The set of NqrB residues 155–160 is of high importance to the binding modes of the simulated ligands; these residues comprise a helix turn forming the central area of the studied binding pocket (Figures 1 and 2). In our simulations, NqrB‐V155, NqrB‐F159, and NqrB‐F160 were frequently identified as interacting residues due to the positioning of their side chain groups. The side chains of these residues point toward the binding pocket, and all are positioned to establish interactions with the simulated ligands (Figure 5). Residues NqrB‐N156 and NqrB‐E157, while highly conserved (Figure 6), establish interactions with the simulated ligands via the backbone as opposed to the side chain. The R‐groups of these residues point away from the binding pocket and are otherwise engaged in maintaining inter‐ and intra‐chain interactions, stabilizing the structure of the pocket. NqrB‐E157 is specifically situated between the basic residues NqrB‐K54 and NqrA‐R372, and its complete conservation is likely due to its importance in maintaining the relative position of these positively charged salt‐bridge partners. Visualization also shows that NqrB‐N156 and NqrB‐E144 play an analogous role in the establishment of binding interactions. While NqrB‐E144 established hydrogen bonding interactions for nearly all simulated frames of HQNO, an analogous interaction was found in one simulation of korormicin‐A. Visualization of korormicin‐A trajectories showed that the side chain of NqrB‐E144 is also engaged in a salt‐bridge interaction with NqrB‐K191. When the side chain of NqrB‐E144 fluctuates away from the binding pocket toward the salt‐bridge partner, the backbone oxygen of NqrB‐N156 occupied the surface of the binding pocket, which was previously occupied by the side chain of NqrE‐144. NqrB‐E144 (via the R‐group) and NqrB‐N156 (via the backbone) thus likely play analogous roles in the establishment of binding interactions.

Residues NqrA‐W337, NqrB‐K54, and NqrB‐M57 comprise another motif of the binding pocket capable of interacting with the warhead of the simulated ligands. Unlike residues NqrB‐155‐160, NqrB‐54‐58 form one complete helix turn adjacent to the binding pocket, thus occluding their backbone atoms from interaction with the ligands. NqrA‐W337 sits adjacent to this helix turn and forms interactions with the warheads of the simulated ligands. While hydrophobic contacts were most typically observed between NqrA‐W337 and the simulated ligands, simulation of aurachin‐D42 also demonstrates the possibility of W337 to π‐stack with bicyclic compounds.

The complementary nature of MD simulations and static structure methods is of the utmost value in evaluating the frequency and quality of molecular interactions. Static structures provide informative starting points for MD simulations, and MD simulations provide quantitative assessments of interactions which are only classified binarily in static structures. Analysis of only the crystallographic and Cryo‐EM structures related to this work (8EVU, 8EW3, 8A1U [29], 8A1V [29], 8A1W [29], 8A1Y [29], 7XK6 [9], and 7XK7 [9]) may mislead researchers on the prevalence of interactions between NQR and the simulated ligand/inhibitors. For example, only a limited number of the aforementioned structures (8EVU and 8EW3) determine the hydrogen bond between NqrB‐K54 and ubiquinone. Our simulations expand understanding of the binding of inhibitors to this motif by demonstrating that HQNO and korormicin‐A also accept a hydrogen bond from NqrB‐K54, which is not observed in the respective static structures (8A1Y and 7XK7). Additionally, in none of the published structures is it evident that NqrA‐W337 may establish interactions with the inhibitors. Our data show that this residue transiently forms π‐stacking interactions with the ligands, in particular with HQNO. Our simulations also elucidate a number of transient hydrophobic contacts with semiconserved residues NqrB‐L33, NqrB‐F137, NqrB‐V145, and NqrB‐V155, which are absent in the Cryo‐EM structures.

In addition to a high resemblance between the dominant pose of simulations to experimentally determined structures (Figure 2), enzymatic analysis corroborated many of the interactions determined in silico in this study. For instance, NqrB‐E144 was identified here as a hydrogen bonding partner (via the side chain) for the binding of HQNO and korormicin‐A. In the study by Ito et al. [65], the E144C mutation of *Vc* NQR severely diminished the inhibitory effect of HQNO, aurachin‐D42, and korormicin‐A. NqrB‐K54 was identified here as a promiscuous hydrogen bonding partner for ubiquinone‐4, HQNO, and korormicin‐A. Ishikawa et al. [66] observed that the mutation K54A severely diminished the activity of NQR. Moreover, this group also identified water‐mediated hydrogen bonding of NqrB‐K54 to aurachin‐D42 and korormicin‐A via Cryo‐EM [9]. While water‐mediated hydrogen bonds are not analyzed here, direct hydrogen bond donation by NqrB‐K54 to each ligand other than aurachin‐D42 was observed. Moreover, Hau et al. [29] identified potential hydrogen bonding between Ubiquinone‐1/Ubiquinone‐2 with the carbonyl backbone oxygen of NqrB‐N156 by Cryo‐EM. While this interaction was not seen in this study, hydrogen bond acceptance by the same oxygen was observed in simulations of korormicin‐A.

Among the NQR residues determined here to be important interaction partners to the simulated ligands and inhibitors, NqrB‐V145 and NqrB‐V155 have not been mutated by ourselves or others. These two residues are adjacently located near the “entrance” of the binding pocket, on the opposite side of NqrA‐W337. NqrB‐V145 is highly conserved and NqrB‐V155 is less conserved, albeit the latter position always carries a small hydrophobic group (Figure 6). Mutation of these residues to typical mutagenesis targets, such as alanine or glycine, is unlikely to significantly impact binding kinetics. However, mutation of NqrB‐V155 to a large bulky group may lower the activity of NQR by occluding the binding site “entrance.” Additionally, NqrB‐V155 is positioned at the start of a critical series of residues NqrB 155–160. Mutation of this residue to a large aromatic residue may not only occlude ubiquinone from binding but disrupt proper formation of the binding pocket altogether—completely disrupting reduction of ubiquinone at the studied site.

## 5 Conclusion

NQR is a viable antibiotic target due to its importance for supporting energy‐consuming reactions and absence in human cells. Here, an analogue of the native ligand (ubiquinone‐4) and three known inhibitors (HQNO, aurachin‐D42, and korormicin‐A) are simulated to elucidate important interactions conferring inhibition. Subunit B residues K54, F137, E144, V145, V155, E157, G158, F159, and F160 were identified as establishing either hydrogen bonding interactions or hydrophobic interactions with at least three of these ligands for significant portions of the respective simulations. These residues, as well as others identified in this study, represent important structural motifs that should be targeted in the formation of structure–activity relationships for novel antibiotics inhibiting NQR.

## Author Contributions

**Joseph. A. DePaolo‐Boisvert:** investigation, methodology, visualization, writing – original draft, writing – review and editing, validation, formal analysis. **Karina Tuz:** conceptualization, funding acquisition, project administration, writing – review and editing, writing – original draft, supervision. **David. D. L. Minh:** methodology, visualization, supervision, writing – review and editing. **Oscar X. Juarez:** conceptualization, funding acquisition, writing – original draft, writing – review and editing, validation, formal analysis, project administration, supervision.

## Conflicts of Interest

The authors declare no conflicts of interest.

## Supporting information


**Data S1:** Structures of studied ligand/inhibitors; RMSD and RMSF of all simulations; all calculated distances.

## Data Availability

The data that support the findings of this study are available on request from the corresponding author. The data are not publicly available due to privacy or ethical restrictions.
