# Supplementary material for: Molecular Dynamics Analysis of Inhibitor Binding Interactions in the Vibrio cholerae Respiratory Complex NQR
Source: Proteins. 2025 Sep 29;94(2):649–59. doi: 10.1002/prot.70036 (PMC12779168; doi:10.1002/prot.70036)
Supplement: Supplementary file 1 — Data S1: Structures of studied ligand/inhibitors; RMSD and RMSF of all simulations; all calculated distances. [file PROT-94-649-s001.docx]

**Supplemental Information for: Molecular Dynamics Analysis of Inhibitor Binding Interactions in the *Vibrio cholerae* Respiratory Complex NQR**

**Joseph. A. DePaolo-Boisvert^1^, Karina Tuz^2^, David. D. L. Minh^1^, and Oscar Juarez*^2^**1 – Department of Chemistry, Illinois Institute of Technology, Chicago, IL
2 – Department of Biology, Illinois Institute of Technology, Chicago, IL
* - Author to whom correspondence should be addressed. Pritzker Science Center, 3101 S. Dearborn St., Chicago, IL. 60616. E-mail: [ojuarez@iit.edu](mailto:ojuarez@iit.edu), Phone: 312-567-3992


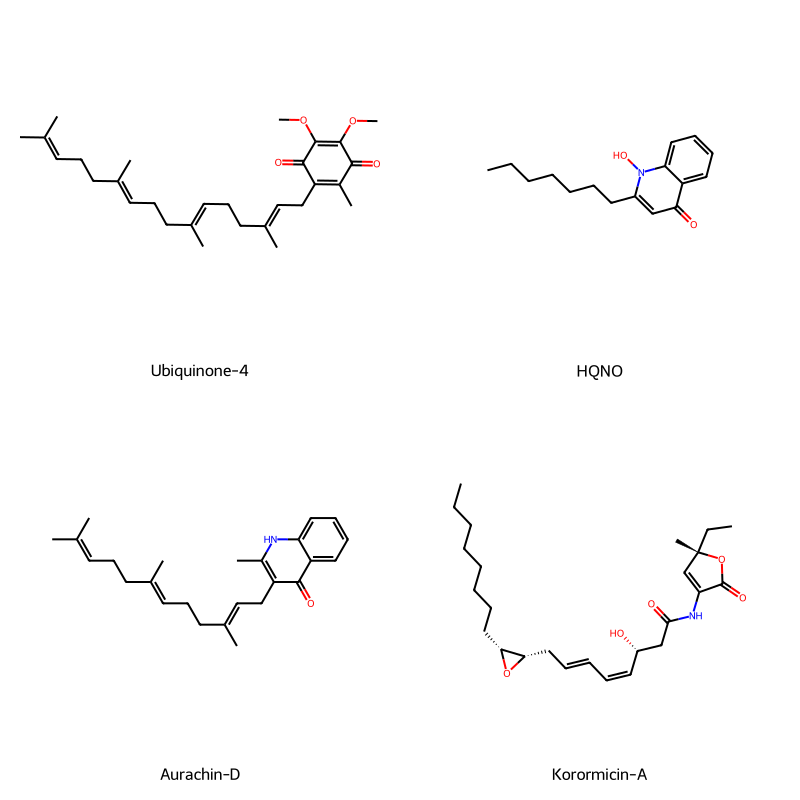


**Figure S1:** Structures of the ligands used in this study.


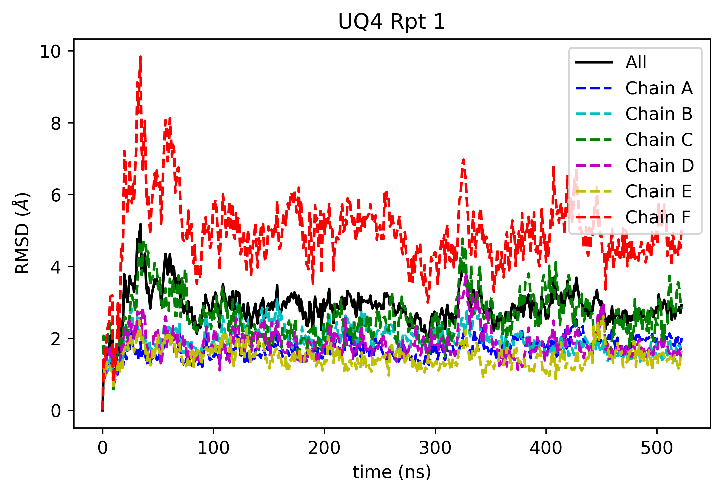

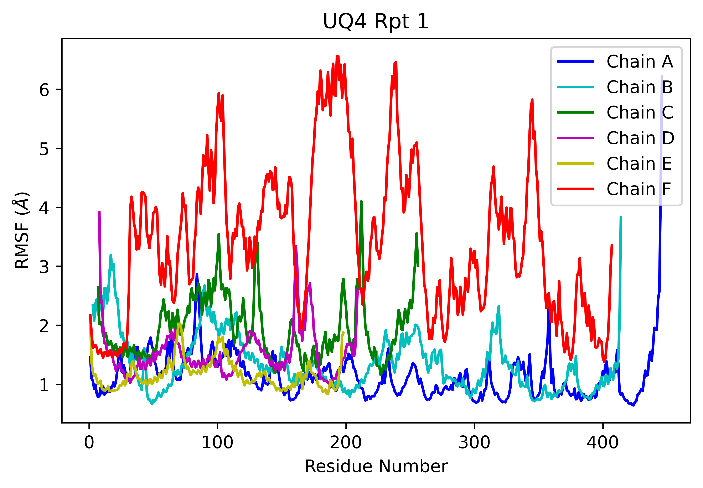


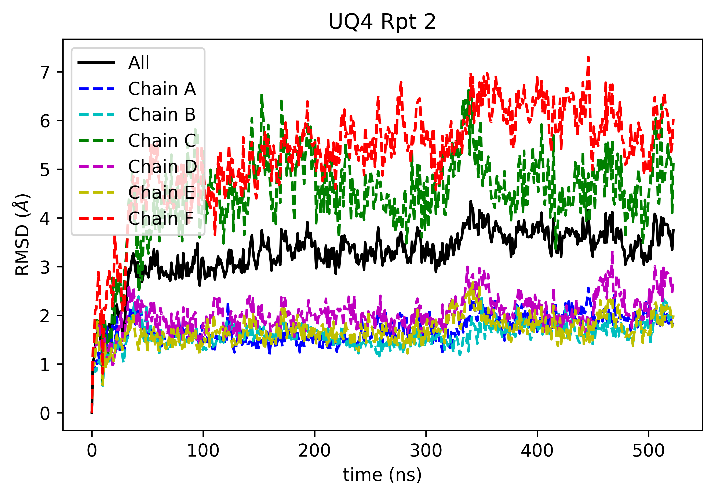

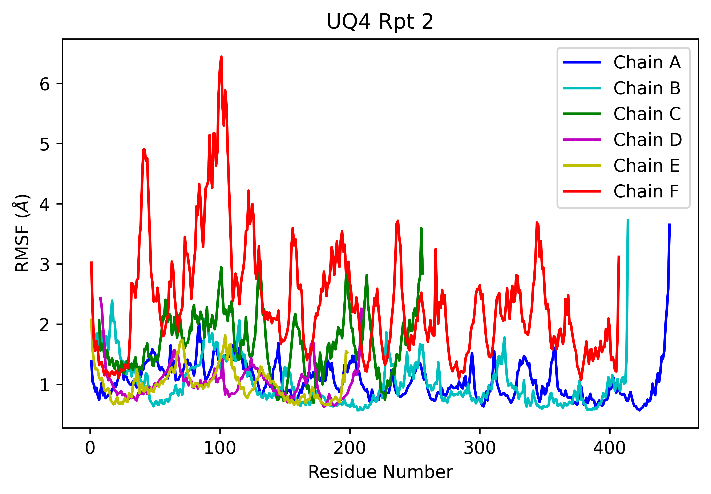


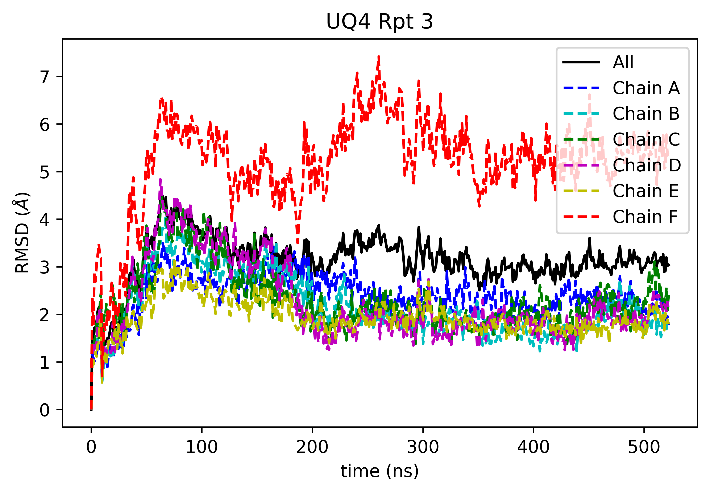

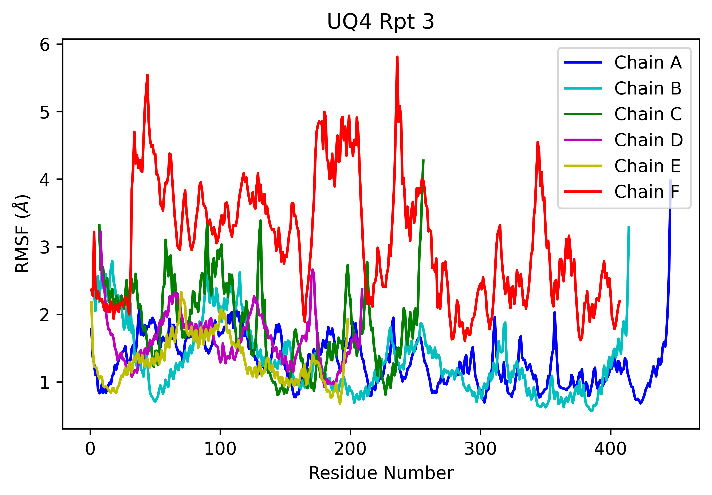


**Figure S2A:** RMSD (Left column) and RMSF (Right column) analyses of triplicate simulations with Ubiquinone-4 as the ligand. Top row: Replicate 1; Middle row: Replicate 2; Bottom row: Replicate 3


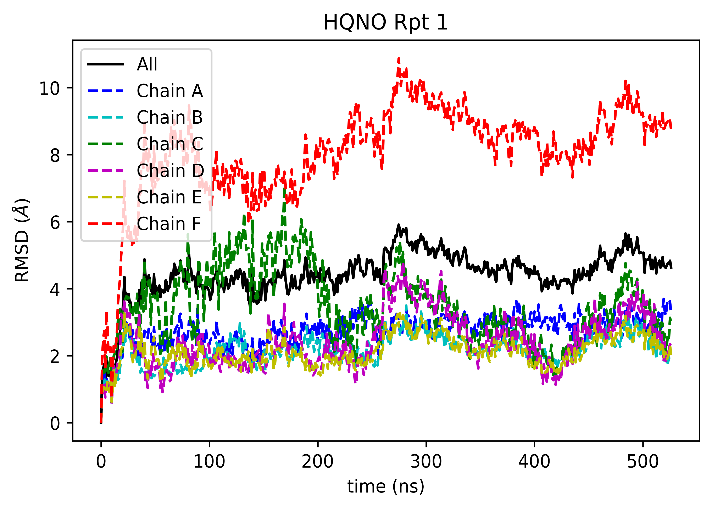

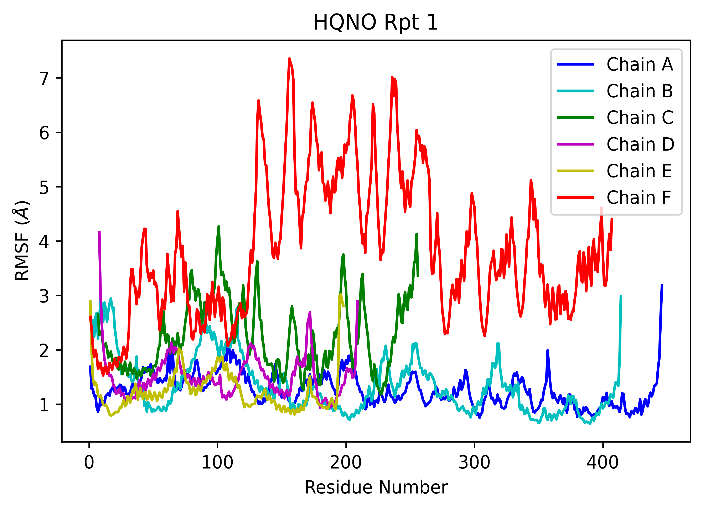

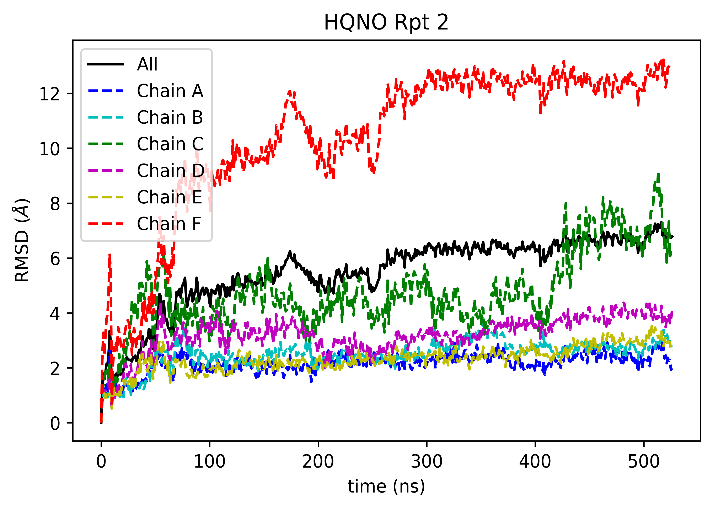

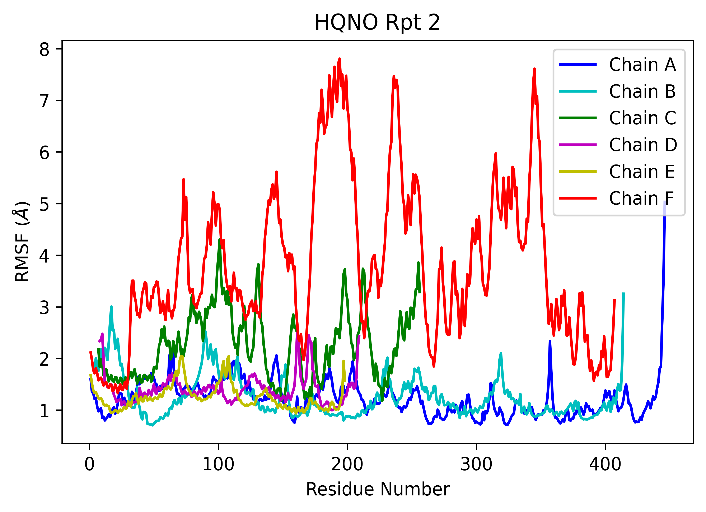

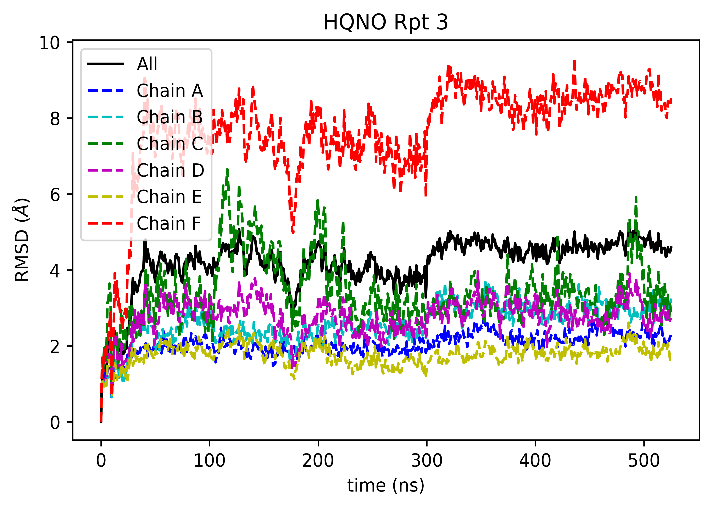

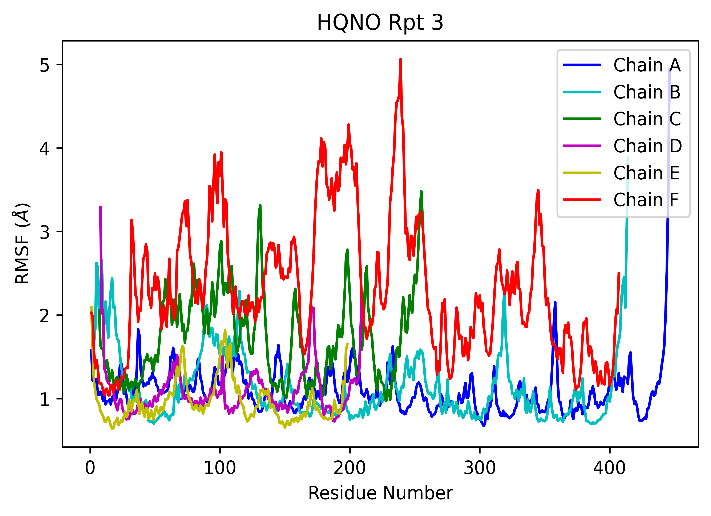


**Figure S2B:** RMSD (Left column) and RMSF (Right column) analyses of triplicate simulations with HQNO as the ligand. Top row: Replicate 1; Middle row: Replicate 2; Bottom row: Replicate 3


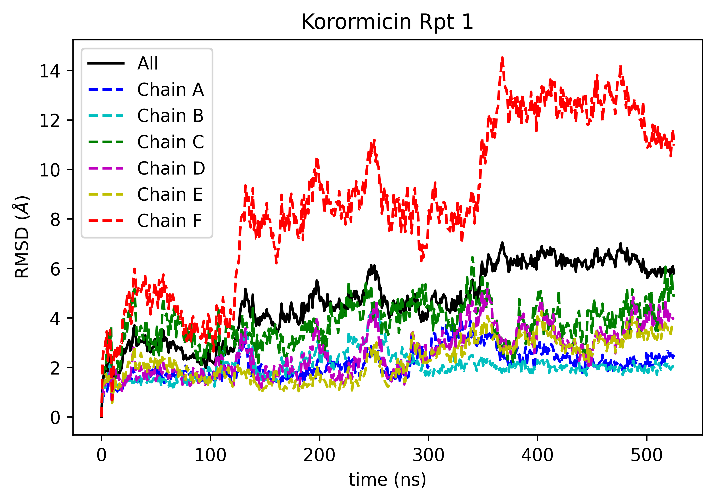

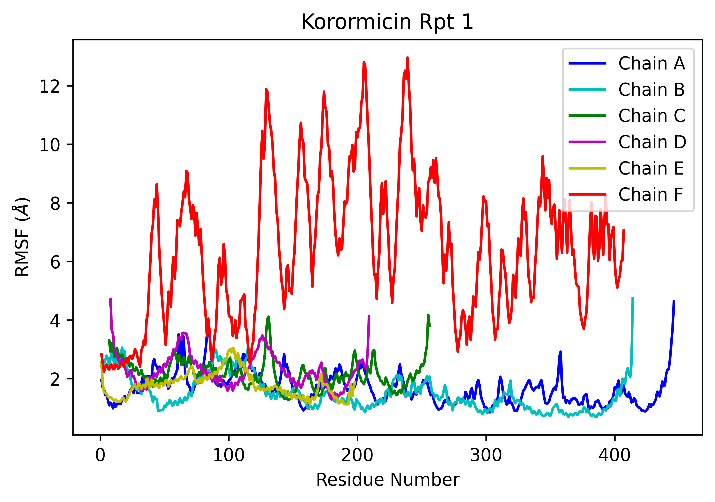

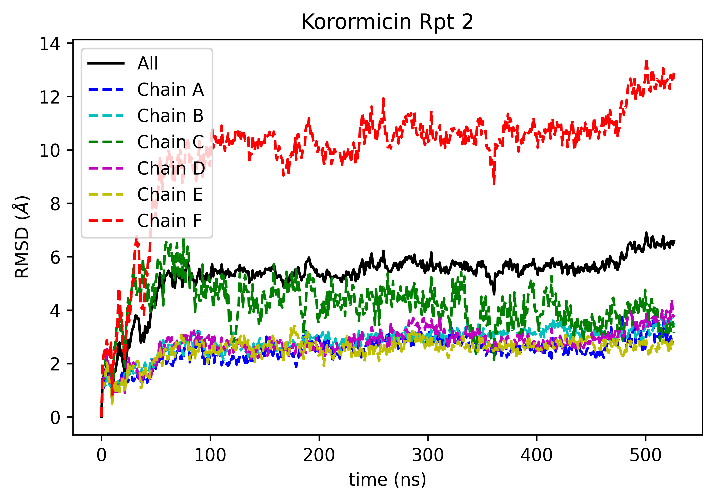

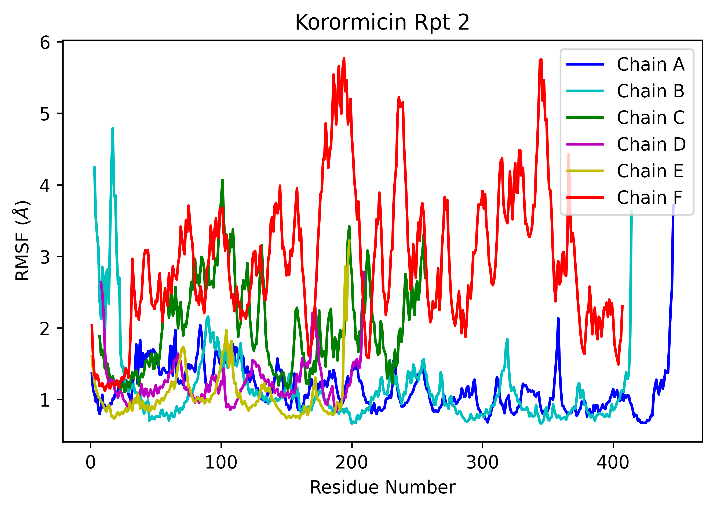

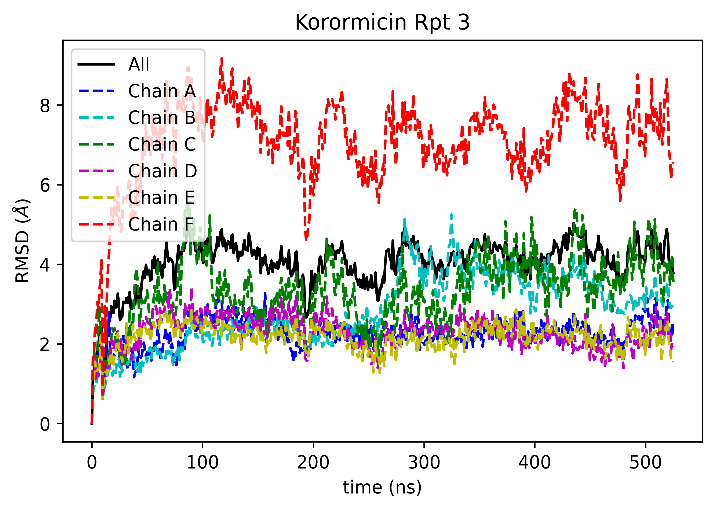

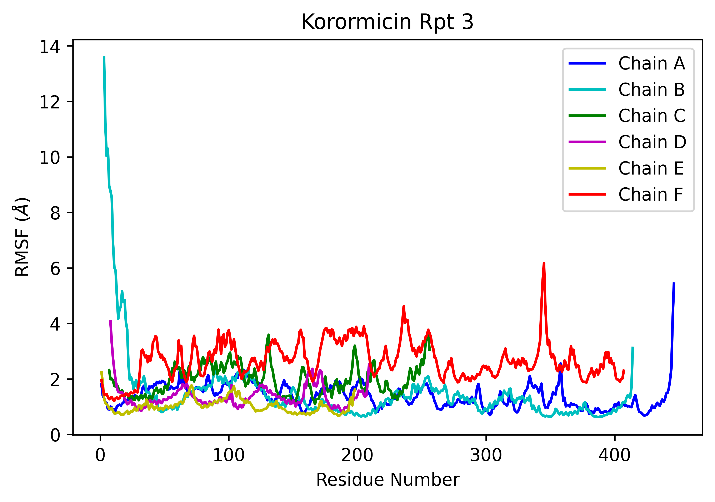


**Figure S2C:** RMSD (Left column) and RMSF (Right column) analyses of triplicate simulations with Korormicin-A as the ligand. Top row: Replicate 1; Middle row: Replicate 2; Bottom row: Replicate 3


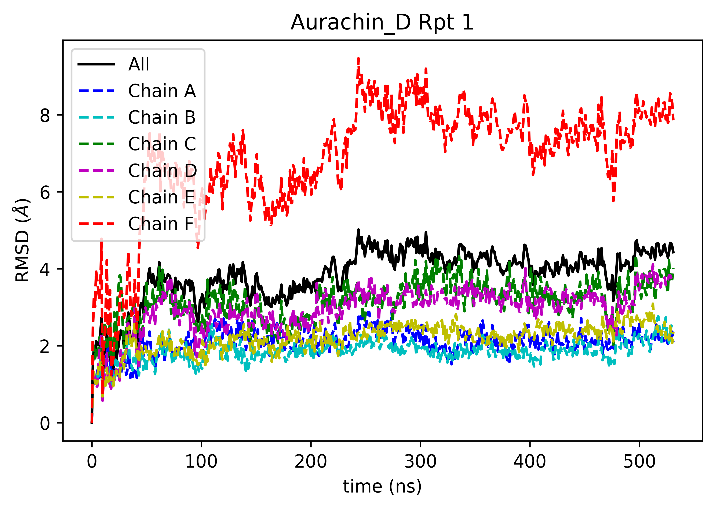

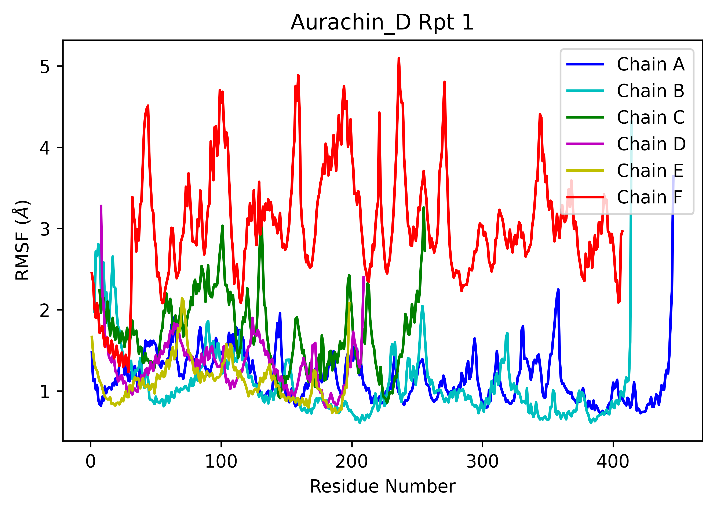

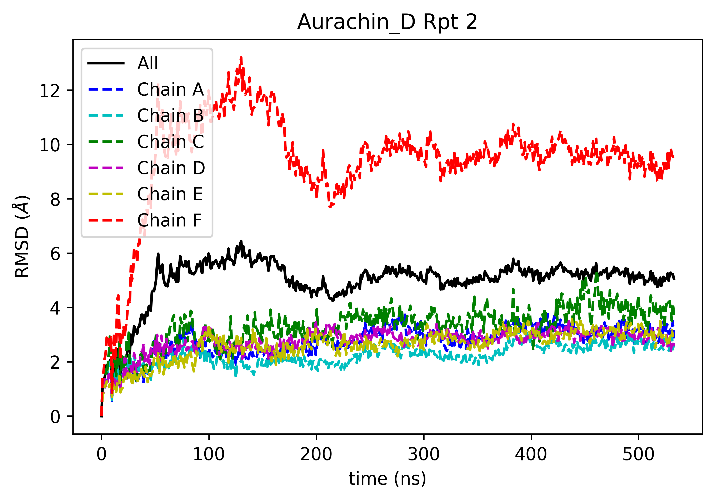

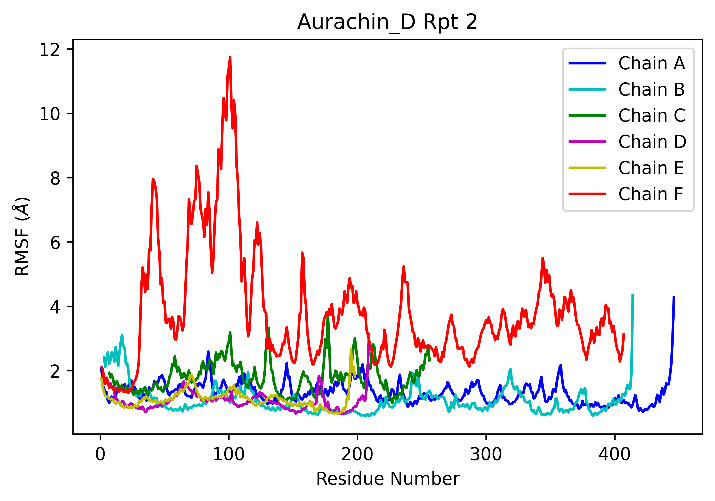

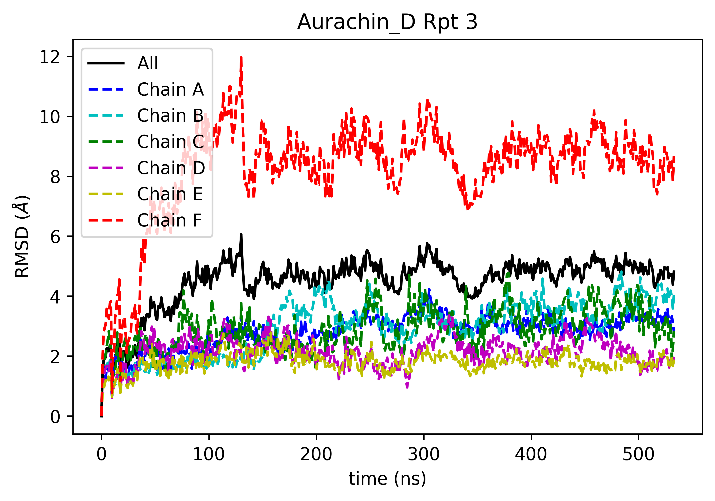

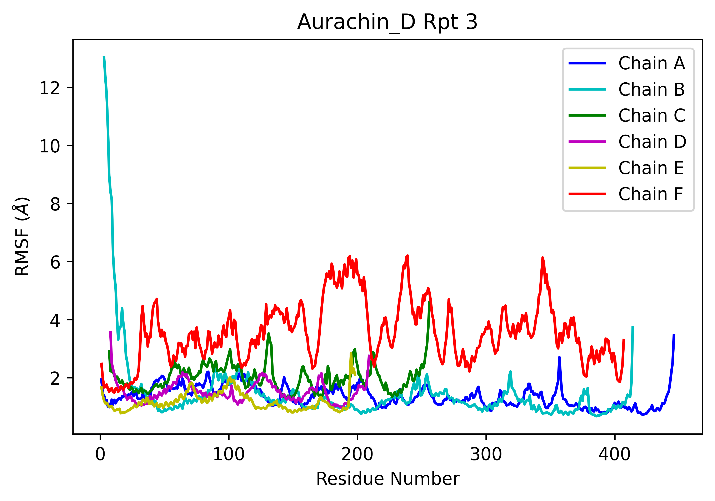


**Figure S2D:** RMSD (Left column) and RMSF (Right column) analyses of triplicate simulations with Aurachin-D as the ligand. Top row: Replicate 1; Middle row: Replicate 2; Bottom row: Replicate 3


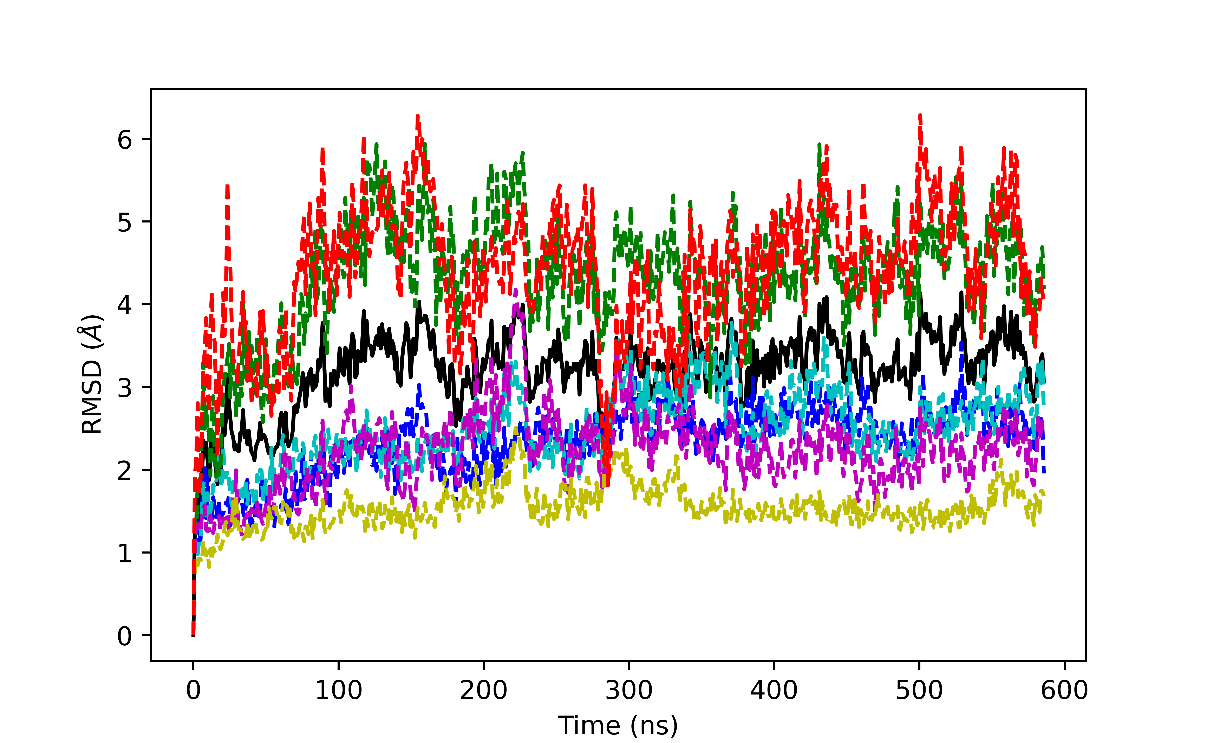

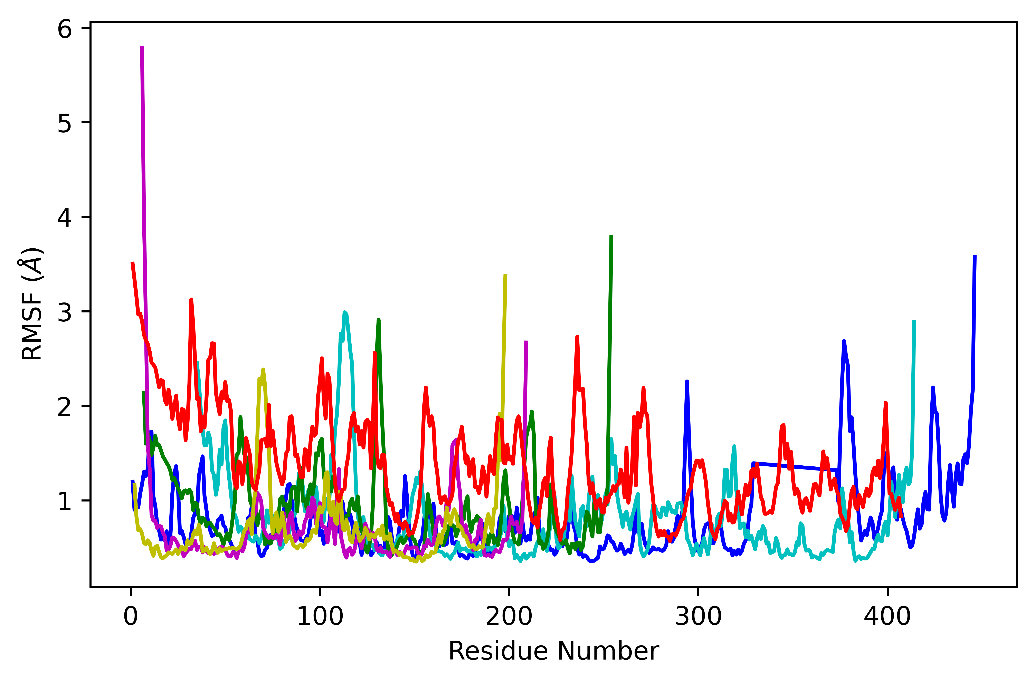


**Figure S2E:** Molecular dynamics simulations based on the 8ACY structure. RMSD (Upper) and RMSF (Lower) of 8ACY validation simulation. Black: all alpha carbon average, Dark Blue: NqrA, Light Blue: NqrB, Green: NqrC, Magenta: NqrD, Yellow: NqrE, Red: NqrF.


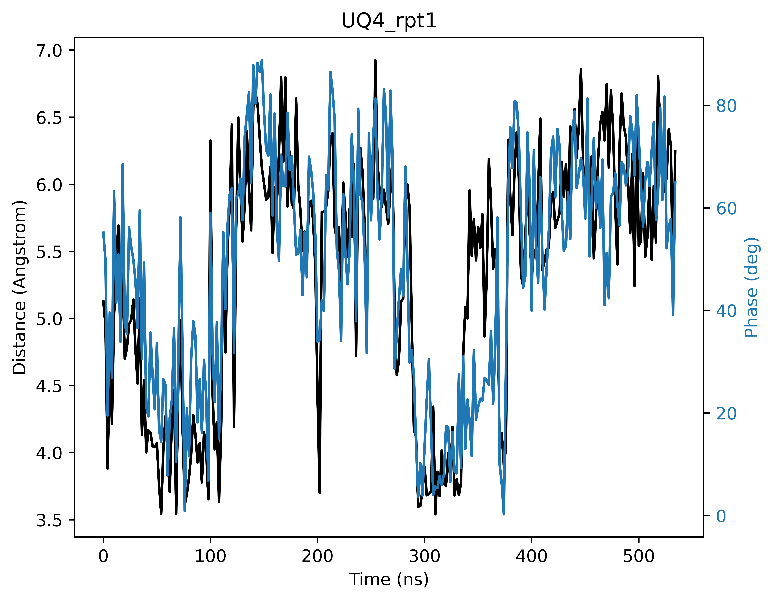

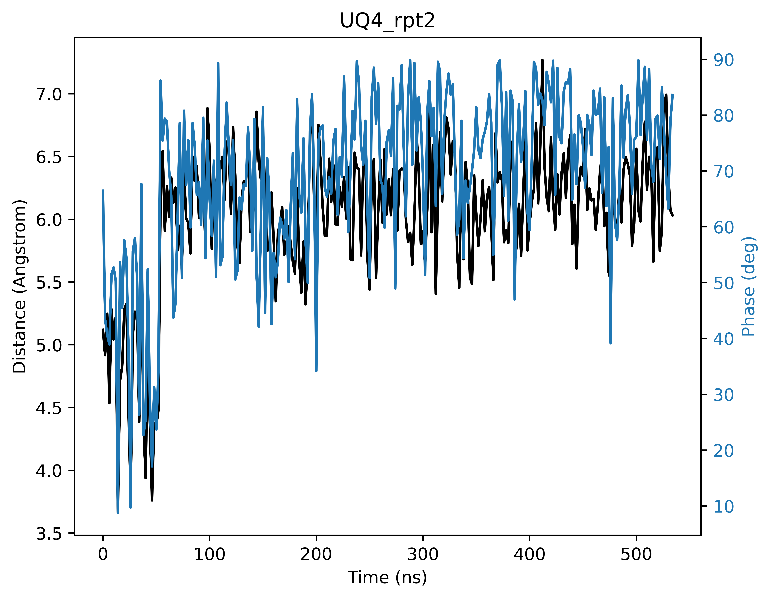

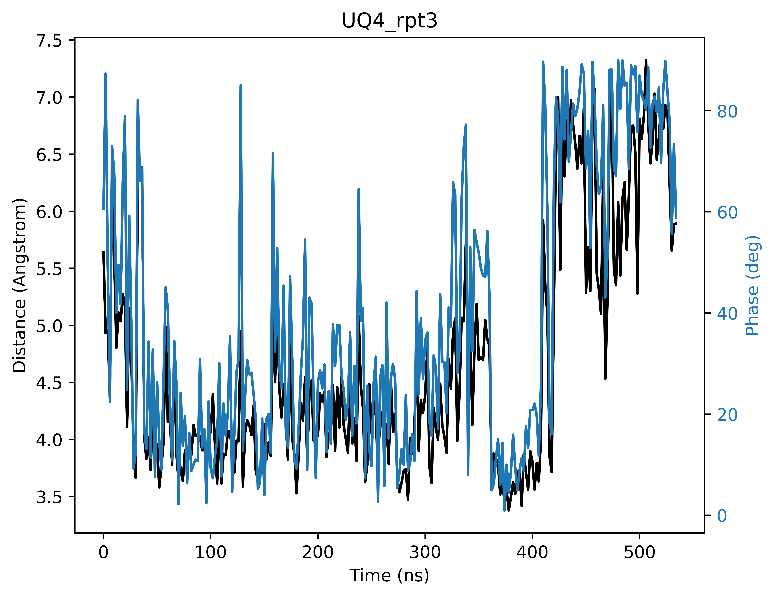


**Figure S3A:** Distance (black) and phase angle (blue) between the side chain heavy atoms of PHE-160-B and heavy atoms of the Ubiquinone-4 warhead. Upper Left – replicate 1; Upper Right – replicate 2; Lower Left – replicate 3.


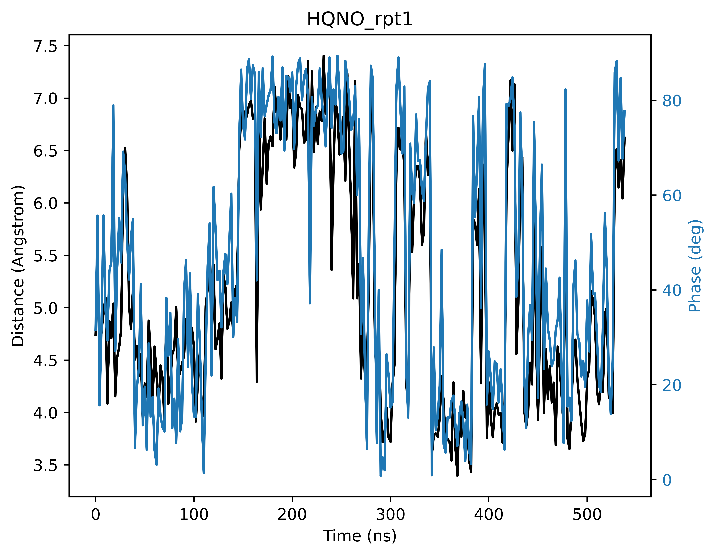

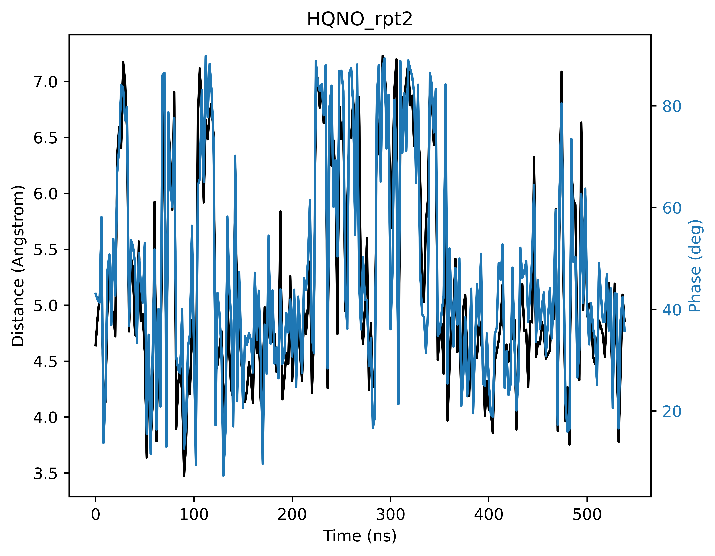

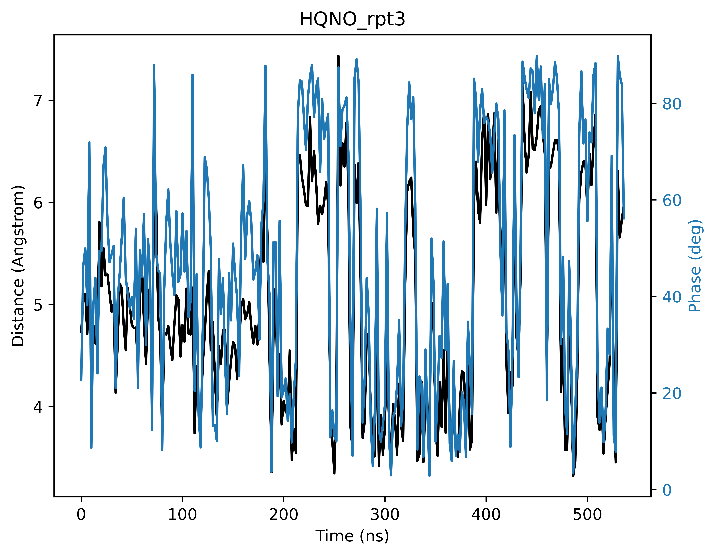


**Figure S3B:** Distance (black) and phase angle (blue) between the side chain heavy atoms of PHE-160-B and heavy atoms of the HQNO warhead. Upper Left – replicate 1; Upper Right – replicate 2; Lower Left – replicate 3.

**
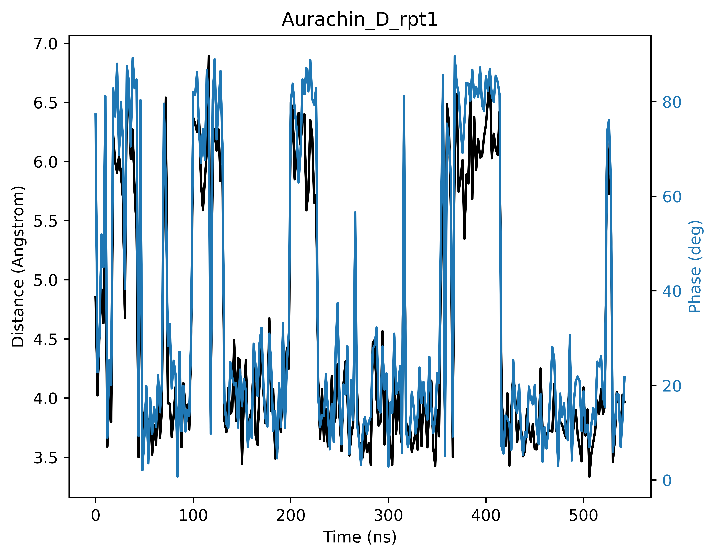

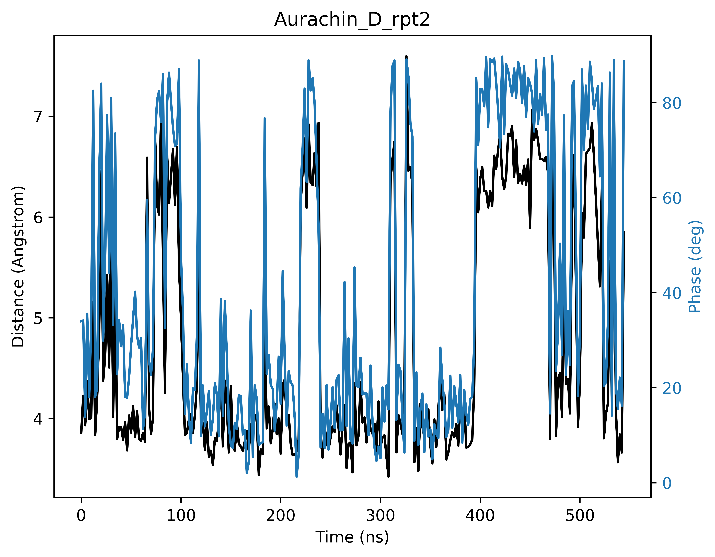

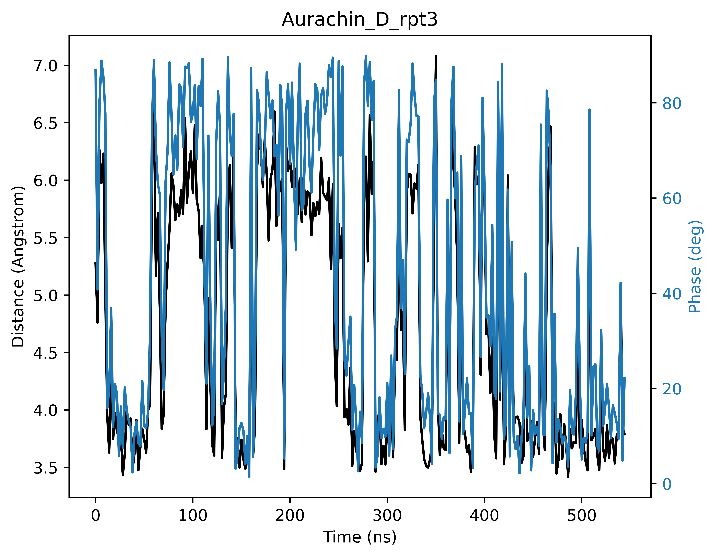
**

**Figure S3C:** Distance (black) and phase angle (blue) between the side chain heavy atoms of PHE-160-B and heavy atoms of the Aurachin-D warhead. Upper Left – replicate 1; Upper Right – replicate 2; Lower Left – replicate 3.


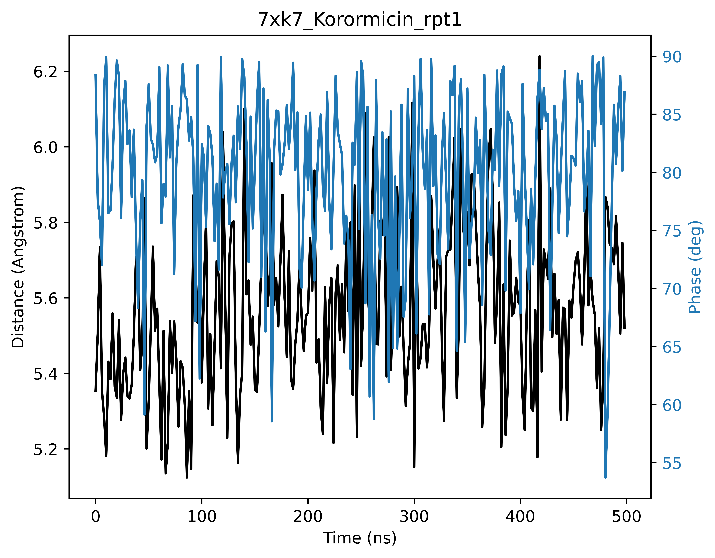

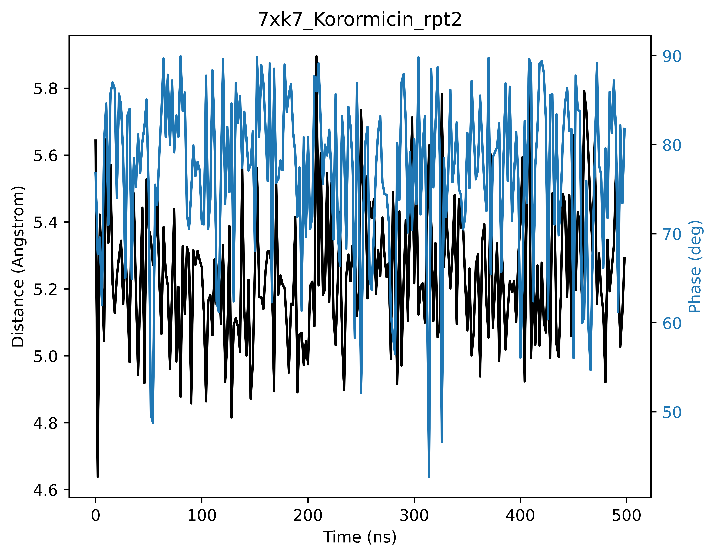

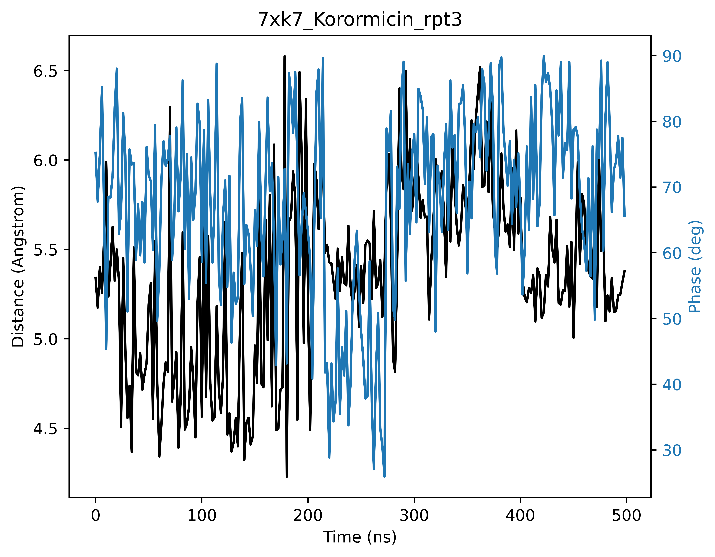


**Figure S3D:** Distance (black) and phase angle (blue) between the side chain heavy atoms of PHE-160-B and heavy atoms of the Korormicin warhead. Upper Left – replicate 1; Upper Right – replicate 2; Lower Left – replicate 3.


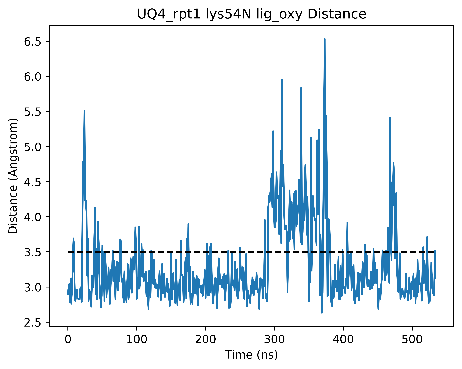

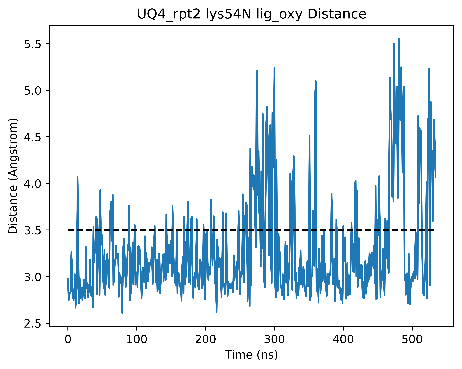

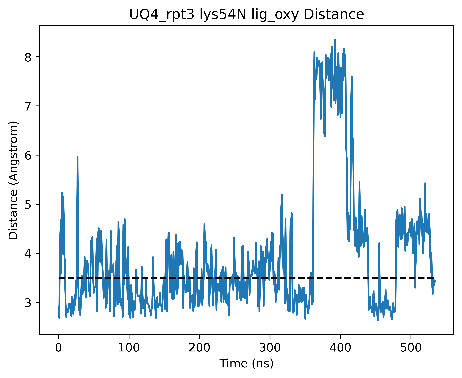


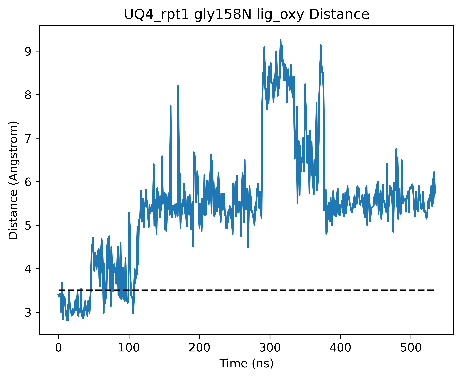

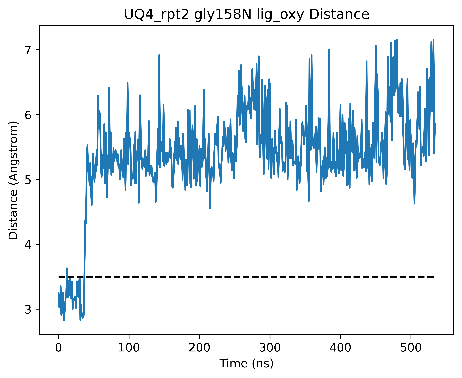

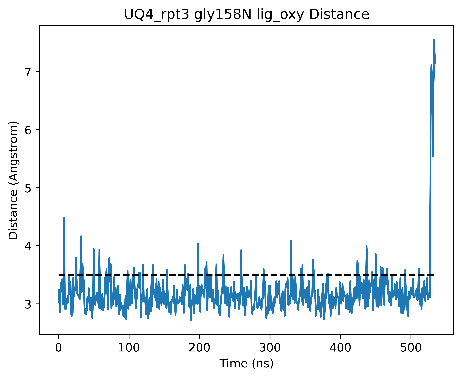


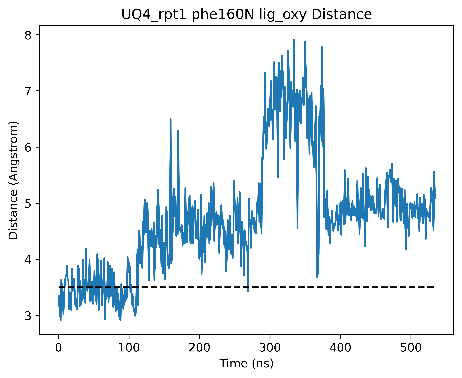

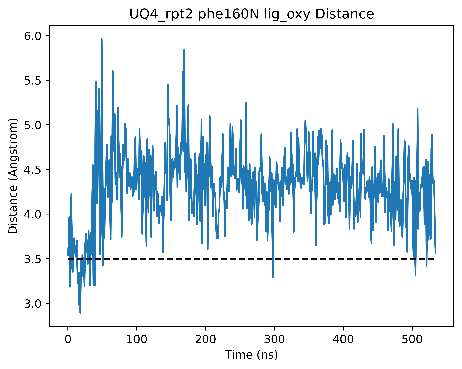

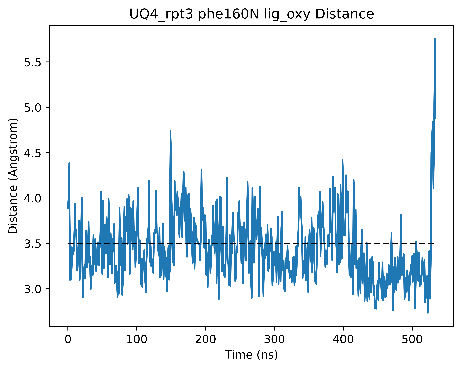


**Figure S4A:** Hydrogen bond distances (Blue) during simulations of Ubiquinone-4. Dashed black line indicates the distance cutoff for detection by ProLIF. Left Column: Replicate 1; Middle Column: Replicate 2; Right Column: Replicate 3.
Distances are plotted between: Top row – R-group nitrogen of LYS-54-B to the closest oxygen of the ligand. Middle row – Backbone nitrogen of GLY-158-B to the closest oxygen of the ligand. Bottom row – Backbone nitrogen of PHE-160-B to the closest oxygen of the ligand.


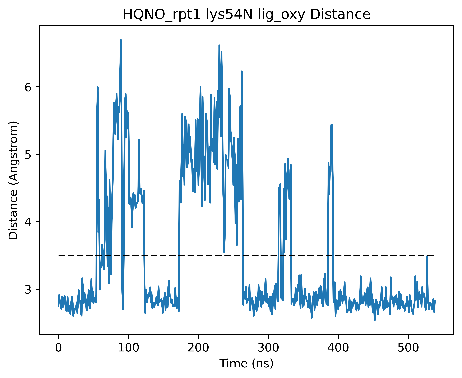

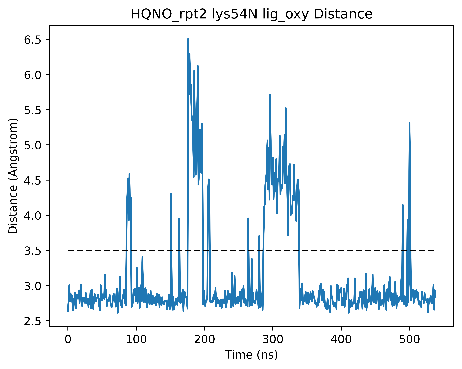

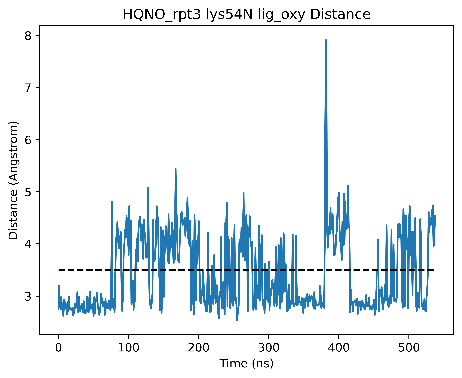

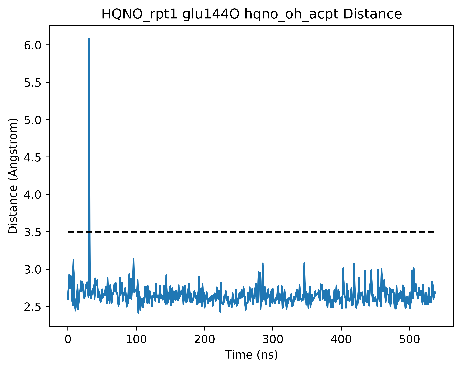

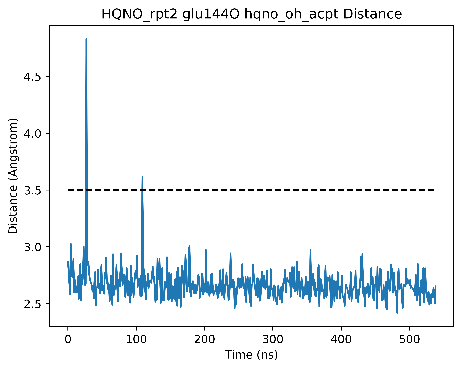

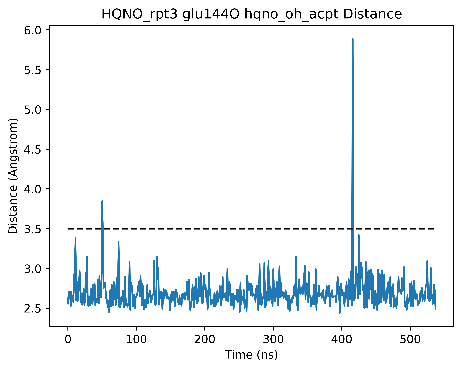

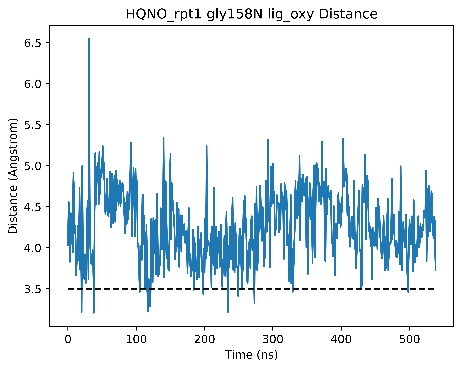

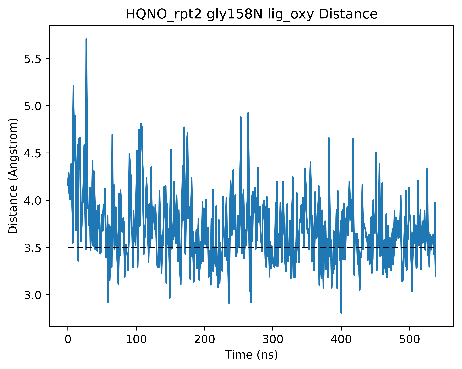

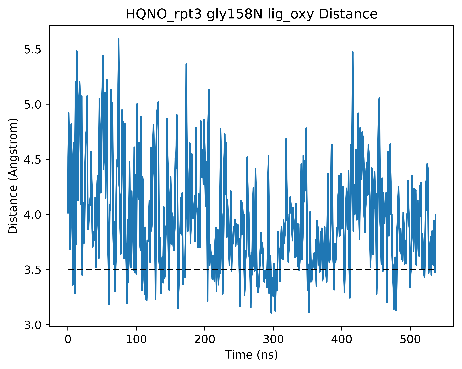


**Figure S4B:** Hydrogen bond distances (Blue) during simulations of HQNO. The dashed black line indicates the distance cutoff for detection by ProLIF. Left Column: Replicate 1; Middle Column: Replicate 2; Right Column: Replicate 3. Distances are plotted between: Top row – R-group nitrogen of LYS-54-B to the closest oxygen of the ligand. Middle row – Hydroxide oxygen of HQNO to the closest oxygen of the GLU-144-B R-group. Bottom row – Backbone nitrogen of GLY-158-B to the closest oxygen of the ligand.


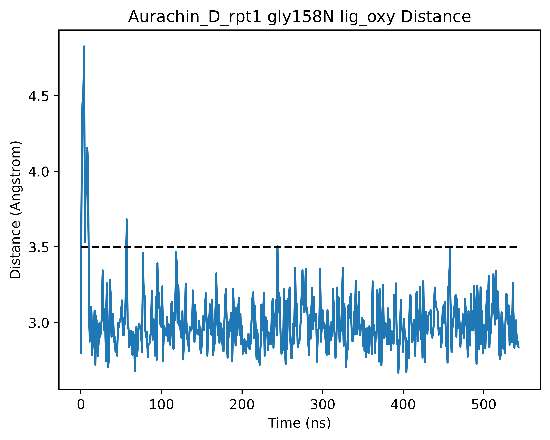

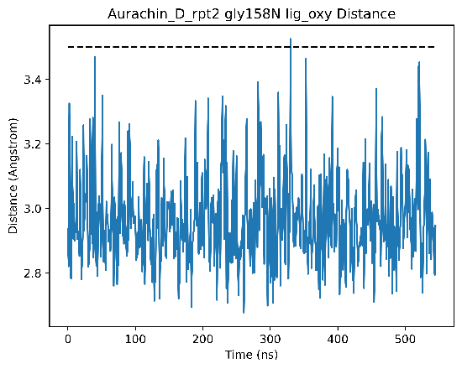

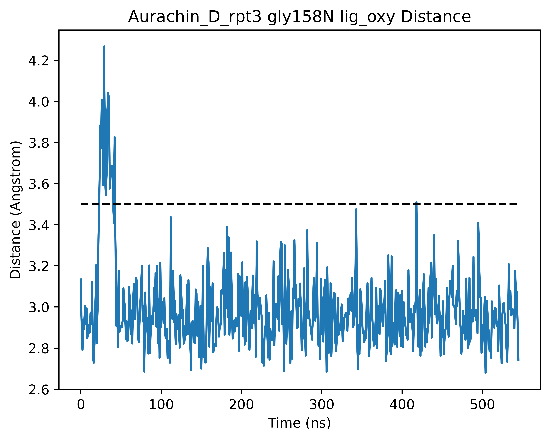

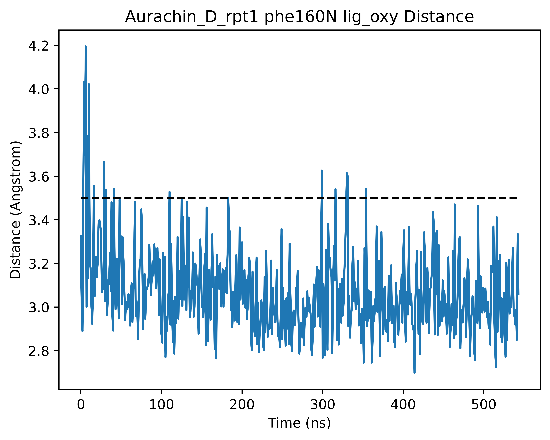

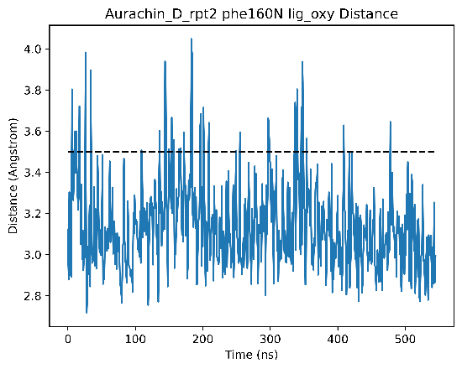

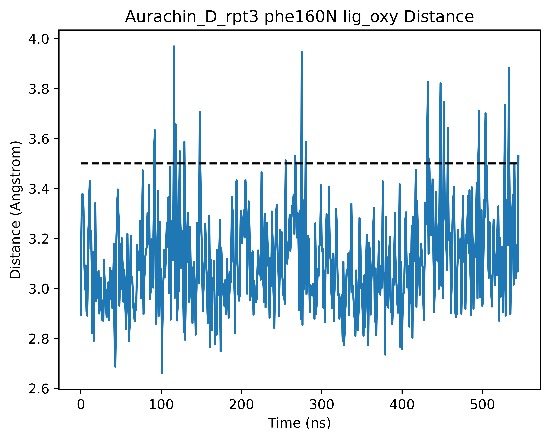


**Figure S4C:** Hydrogen bond distances (Blue) during simulations of Aurachin-D. The dashed black line indicates the distance cutoff for detection by ProLIF. Left Column: Replicate 1; Middle Column: Replicate 2; Right Column: Replicate 3. Distances are plotted between: Top row - Backbone nitrogen of GLY-158-B to the closest oxygen of the ligand. Bottom row - Backbone nitrogen of PHE-160-B to the closest oxygen of the ligand.


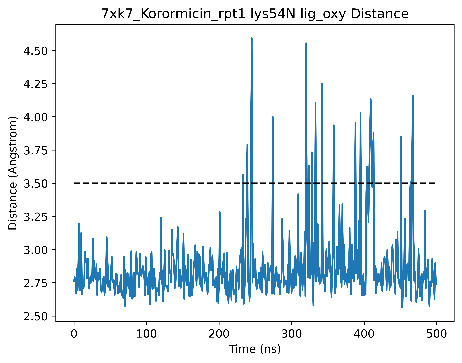

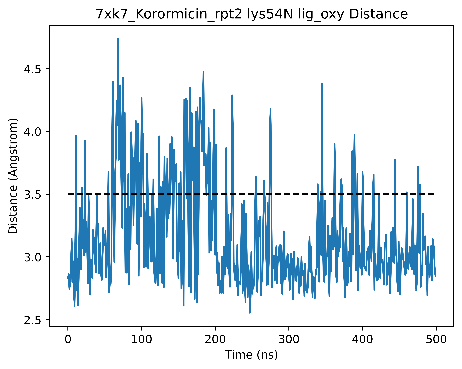

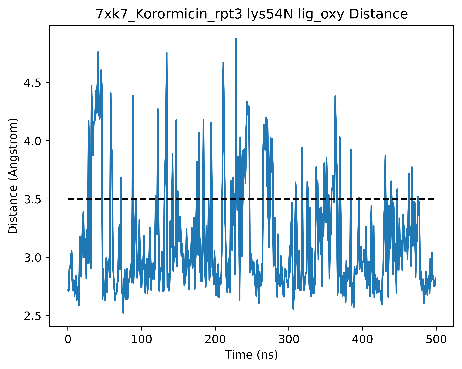

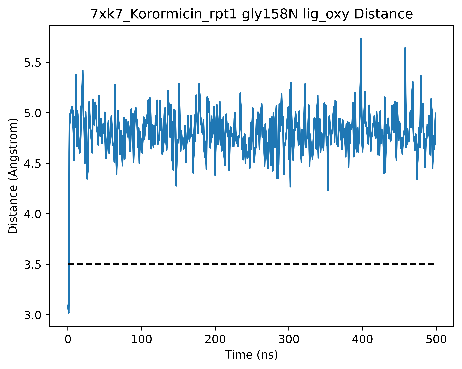

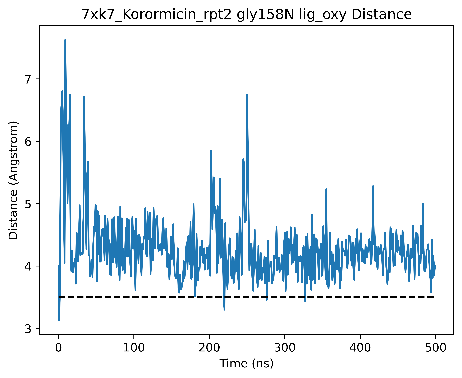

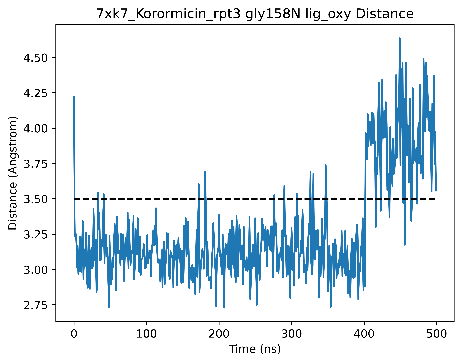

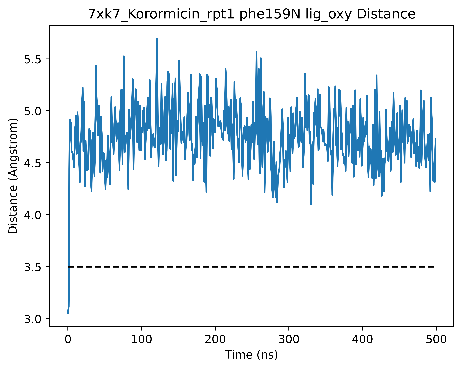

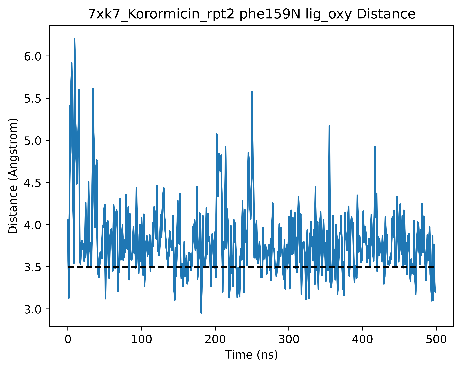

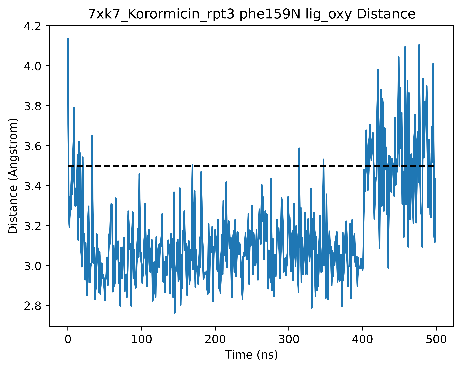


**Figure S4D:** Hydrogen bond distances (Blue) during simulations of Korormicin. The dashed black line indicates the distance cutoff for detection by ProLIF. Left Column: Replicate 1; Middle Column: Replicate 2; Right Column: Replicate 3. Distances are plotted between: Top row – R-group nitrogen of LYS-54-B to the closest oxygen of the ligand. Middle row – Backbone nitrogen of GLY-158-B to the closest oxygen of the ligand. Bottom row – Backbone nitrogen of PHE-159-B to the closest oxygen of the ligand.


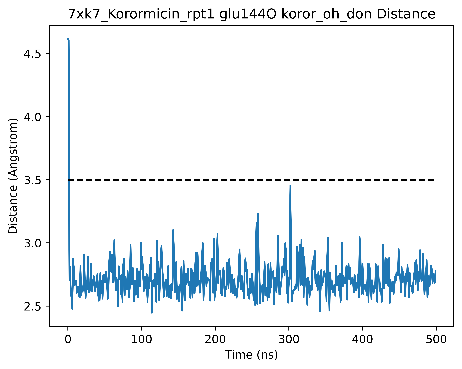

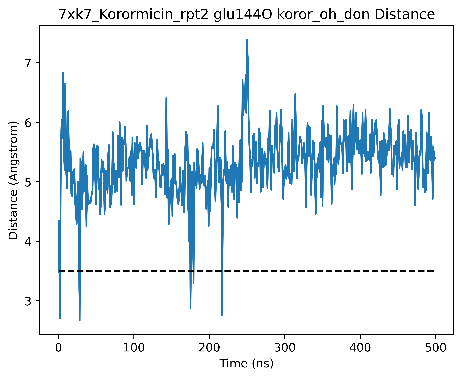

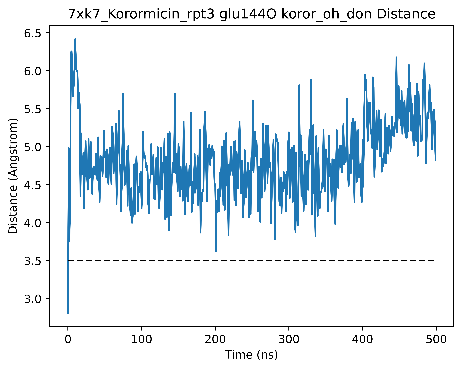

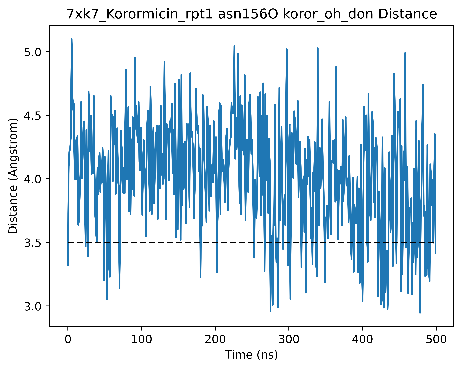

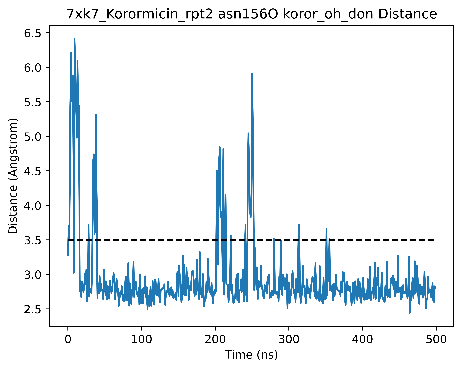

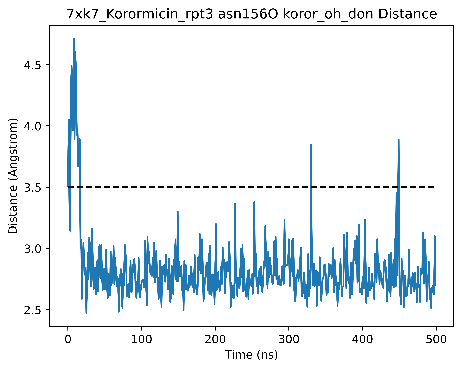

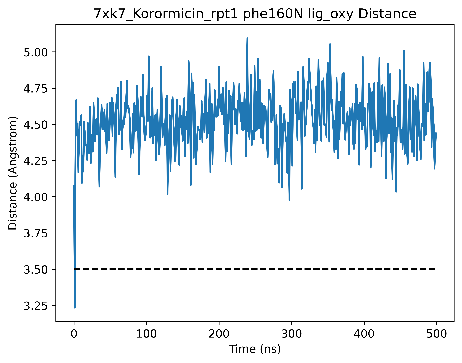

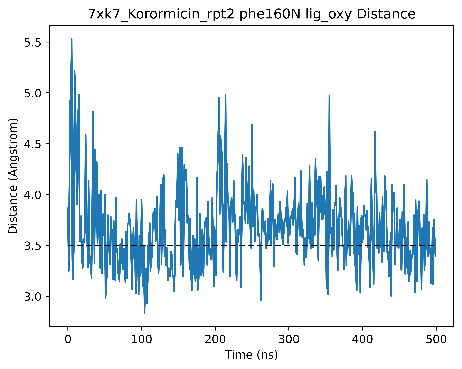

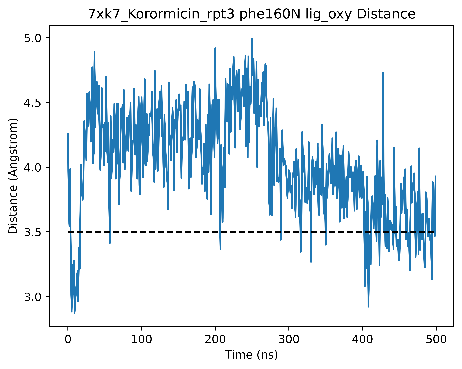


**Figure S4E:** Hydrogen bond distances (Blue) during simulations of Korormicin. The dashed black line indicates the distance cutoff for detection by ProLIF. Left Column: Replicate 1; Middle Column: Replicate 2; Right Column: Replicate 3. Distances are plotted between: Top row – Hydroxide oxygen of Korormicin to the closest oxygen of the GLU-144-B R-group. Middle row – Hydroxide oxygen of Korormicin to the ASN-156-B backbone oxygen. Bottom row – Backbone nitrogen of PHE-160-B to the closest oxygen of the ligand.
